# Designing Molecular Solar Thermal Systems Based on the Paternò–Büchi Reaction Coupled to Enzymatic Energy Release

**DOI:** 10.1002/cssc.202500777

**Published:** 2025-06-16

**Authors:** Marta Delgado‐Gómez, Jesús Reategui Illatopa, Lorenzo Gramolini, Richard López‐Corbalán, Cristina García‐Iriepa, Marco Marazzi

**Affiliations:** ^1^ Universidad de Alcalá Departamento de Química Analítica Química Física e Ingeniería Química Functional Molecular Systems (FuMSys) group Ctra. Madrid‐Barcelona, km. 33,600 Alcalá de Henares 28805 Madrid Spain; ^2^ Universidad de Alcalá Instituto de Investigación Química “Andrés M. del Río” Ctra. Madrid‐Barcelona, km. 33,600 Alcalá de Henares Madrid 28805 Spain

**Keywords:** enzymatic catalysis, molecular modeling, molecular solar thermal systems, Paternò–Büchi reaction, solar energy storage

## Abstract

Molecular solar thermal systems are attracting considerable attention as an alternative to conventional batteries for storing chemical energy, making it possible to use sunlight as external storage input, while releasing the stored energy as heat. Despite such interest, acceptable results are obtained only by modifying the norbornadiene–quadricyclane system, still leaving key issues unsolved. Here, a full storage‐release molecular solar thermal systems cycle based on the Paternò–Büchi reaction is designed, potentially offering a class of compounds with a significantly higher storage density than norbornadiene–quadricyclane. Based on the experimental evidence concerning the viability of their synthesis and photoreactivity, those compounds are repurposed by computationally elucidating the substitution pattern effects on low‐energy isomer's light absorption, followed by high‐energy isomer's photoproduction, including singlet and triplet states involved by the Paternò–Büchi type of reactivity. The thermal conversion back to the initial isomer to release the stored energy is also studied, including a sustainable option by taking advantage of enzymatic activity.

## Graphical abstract

Molecular solar thermal systems urge for innovative building blocks to increase their present limits in storage density and light activation window. Here, a complete computational study of loading and unloading mechanisms for an experimentally inspired system based on the Paternò–Büchi reaction is presented. Unconventional enzymatic reversion is proposed to release the stored energy.

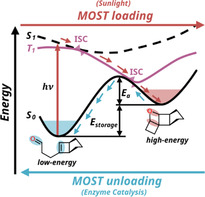

## 1 Introduction

**Figure 1:**
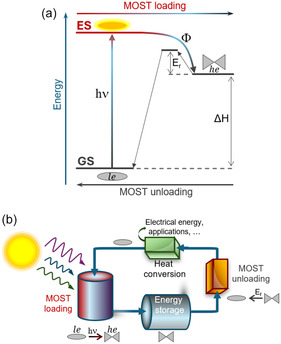
a) Energy‐level diagram elucidating the mechanism of the MOST system: a low‐energy (*le*) isomer absorbs a photon (h*ν*) promoting the vertical transition from the electronic ground state (GS) to the excited state (ES), followed by photochemical formation of the high‐energy (*he*) isomer dictated by a specific quantum yield (*Ф*), thus concluding the MOST loading path. The difference in enthalpy between the two isomers (Δ*H*) corresponds to the energy that the MOST system can store. A GS energy barrier (*E*_r_) determines the lifetime of the high‐energy isomer before releasing the stored energy as heat and concludes the MOST unloading path. b) MOST cycle from the technological perspective.

Molecular solar thermal (MOST) systems are increasingly attracting the attention of the scientific community as a promising concept and emerging technology: if properly designed, the MOST system could store solar energy in the form of chemical energy for a certain amount of time, followed by energy release as heat, when desired.^[^ ^1^ ^]^ Historically, the idea of solar energy storage through molecules dates back to 1909, when solid‐state anthracene photo‐dimerization was first proposed.^[^ ^2^ ^]^ From the chemical standpoint, the MOST functioning mechanism is attractive also for its apparent simplicity (**Figure** **1**a). A low‐energy isomer absorbs solar light photo‐generating a high‐energy isomer, with the associated energy difference corresponding to the stored energy.^[^ ^3^ ^]^ If the high‐energy isomer is thermally and photochemically stable, it can store such energy for a certain amount of time, before energy release is required. This means, in principle, that a suitably designed or modified molecular photoswitch could be considered an appropriate MOST system.^[^ ^4^ ^]^

From the technological standpoint, however, the state of matter should also be considered: solid‐state MOST systems show limitations,^[^ ^5^ ^]^ while liquid‐state MOST systems would be the most indicated for completing a cycle (Figure 1b).^[^ ^6^, ^7^ ^]^ Hence, in the last four decades MOST devices based on a liquid system were mainly proposed,^[^ ^8^ ^]^ through extensive studies of sets of organic and organometallic molecules, with the repurposing aim of identifying good candidates among previously investigated photoswitches. In principle, the following properties are simultaneously required:^[^ ^6^, ^9^, ^10^ ^]^ 1) The absorption spectrum of the low‐energy isomer needs to match, to the greatest possible extent, the solar spectrum in terms of photonic energy (h*ν*). 2) The photochemical path generating the high‐energy isomer has to avoid the formation of byproducts and side products to any extent, hence maximizing the quantum yield (*Ф*).^[^ ^11^ ^]^ 3) A high energy‐storage density (*ρ*_s_), measured as the enthalpy difference between high‐ and low‐energy isomers per unit weight, hence preferring a low molecular mass (*ρ*_s_ = Δ*H*/*M*). 4) A consistently high thermal barrier for the releasing step (*E*_r_, from high‐ to low‐energy isomer) matching the desired storage time. 5) Ideally, the liquid should be pure to maximize the number of MOST chromophores per unit of volume or, at least, it should be soluble in a minimum amount of solvent, this last being more ecologically sustainable if implying an aqueous environment.^[^ ^12^ ^]^

With this list in mind, different types of known photoswitching mechanisms were proposed, sharing the basic design principle based on increasing the structural tension after photon absorption: photo‐ring opening (dihydroazulene/vinylheptafulvene system);^[^ ^13^, ^14^ ^]^ *E*/*Z* photoisomerization, mainly through stilbenes and azobenzenes;^[^ ^15^, ^16^ ^]^ multichromophoric macrocyclic deformation, by coupling azobenzene units; photoisomerization of bimetallic complexes, especially using ruthenium;^[^ ^16^ ^]^ [2 + 2] photocycloaddition, finding in the norbornadiene/quadricyclane (NBD/QC) system the most promising MOST candidate, due to the absence of side products, no photochemical reversion in the solar range, and a considerable stored enthalpy.^[^ ^17^, ^18^, ^19^ ^]^ Nevertheless, although many experimental and theoretical efforts were spent in the last decade to improve the NBD/QC system (mainly including a deep investigation of substitution patterns,^[^ ^9^, ^20^ ^]^ also through theoretical mechanochemistry),^[^ ^21^ ^]^ two main drawbacks are weakening the hope to see them stepping out from chemistry labs to finally reach the technological stage: 1) A consistent portion of the visible light can be absorbed only at the expenses of the energy‐storage density, since acceptor and donor groups need to be introduced, critically increasing the unit weight and 2) solubility issues.

Therefore, novel strategies need to be identified. Recently, in an attempt to increase the stored energy by modifying conventional *E*/*Z* photoswitches, phase‐transition MOST systems were developed, where photoisomerization is coupled to solid–liquid phase change, hence adding the melting enthalpic term to the isomerization one.^[^ ^22^, ^23^ ^]^ Nevertheless, although attractive from the thermodynamic standpoint, this would lead to substantial technological difficulties in finding a device setup allowing the low‐energy isomer to be stored as a solid.

Another strategy that was implemented was the synthesis of NBD‐based oligomers (dimers and trimers), where a central donor building block is covalently linked to NBD‐acceptor units,^[^ ^24^, ^25^ ^]^ making it possible for a single oligomer to accept photons at different energy, thus covering a larger absorption window also using sensitizers,^[^ ^26^ ^]^ although resulting in conjugated oligomers with even higher solubility issues compared to separated chromophores.

In this contribution, we suggest a different strategy. Step back from the repurpose approach that guided almost all the MOST systems proposed until now and came up with different photochemical systems.^[^ ^11^, ^27^ ^]^ More in detail, we show how the Paternò–Büchi reaction^[^ ^28^ ^]^ can be helpful in designing MOST systems with distinctive photochemistry and with higher energy‐storage densities.

Moreover, we propose an alternative strategy to release the stored energy on demand. Usually, organo‐metallic heterogenous catalysts were applied to decrease the thermal barrier required to convert back the high‐energy isomer into the initial low‐energy one. Among others, platinum‐, copper‐, nickel‐,^[^ ^29^ ^]^ and silver‐^[^ ^30^ ^]^ based catalysts were found to facilitate the thermal QC‐to‐NBD reaction, although the underlying mechanisms remain largely elusive. Moreover, the usage of such metals cannot be considered ecologically friendly. Alternatively, the energy release step could be electrochemically triggered at the interface with an electrode, although only a few examples were reported,^[^ ^31^, ^32^ ^]^ mainly due to a limited chemical efficiency caused by unwished side reactions and electrode deterioration.^[^ ^33^ ^]^ Here, we propose a much greener solution: the employment of enzymatic catalysis to revert the Paternò–Büchi reaction.

## 2 Results and Discussion

### 2.1 Design Strategies

**Figure 2:**
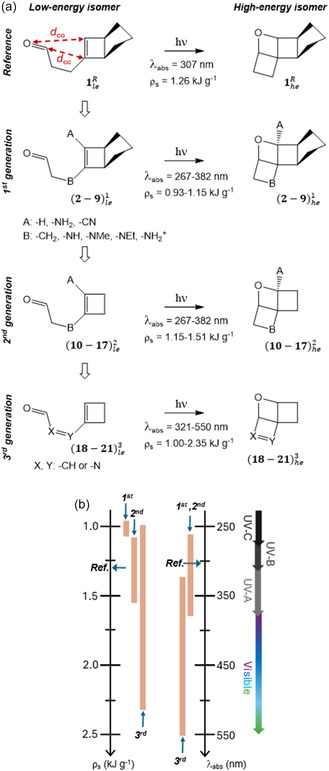
a) Photochemical reactivity studied in this work, including low‐ and high‐energy isomers of the reference system and its three identified generations of compounds, all having in common a Paternò–Büchi reaction with the concomitant formation of a tri‐cyclic photoisomer, through the formation of two *σ*‐bonds identified by the distances *d*_CO_ and *d*_CC_. Each compound is numerically identified, including a subscript for the type of isomer (*le*: low‐energy; *he*: high‐energy) and a superscript for the type of molecule (*R*: reference; 1, 2, 3: each generation). See Table 1 for the definition and numbering of each compound. The calculated maximum absorption wavelengths (*λ*_abs_) and the energy‐storage densities (*ρ*_s_) are given. b) Visual comparison in terms of both *λ*_abs_ and *ρ*_s_ range.

The choice of the type of molecular system to be studied was guided by experimental evidence. A cyclobutene aldehyde bicyclic derivative ($1_{\text{le}}^{\text{R}}$) can be synthesized in a two‐step route characterized by considerable yields, and later irradiated to form a stable oxetane tetracyclic structure ($1_{\text{he}}^{\text{R}}$) via an intramolecular Paternò–Büchi reaction (**Figure** **2**a).^[^ ^34^ ^]^ Such experimental finding was presented by Buendia et al. as a feasible way to prepare unprecedented angular tricyclic oxetane‐containing skeletons. Here, we focus on this building block offering a highly strained high‐energy isomer as a reference to possibly developing a novel MOST system.

The most appealing parameter is the expected high *ρ*_s_ calculated value, 1.26 kJ g^−1^ (see **Table** **1**), compared to 1.00 kJ g^−1^ of NBD/QC, calculated at CASPT2 level^[^ ^17^ ^]^ and experimentally validated.^[^ ^35^, ^36^, ^37^ ^]^ Nevertheless, similarly to the NBD/QC bare (unsubstituted) system, the main drawback of $1_{\text{le}}^{\text{R}}$ for its use as MOST is its absorption spectrum lying in the UV region (maximum at 307 nm). As shown in Figure 2, we developed the principal idea of a Paternò–Büchi reaction based MOST, by envisioning three generations starting from the reference system.

**Table 1:** CASPT2 *S*_0_ → *S*_1_ maximum absorption wavelength (*λ*_abs_), oscillator strength (*f*), and energy‐storage density (*ρ*_s_) calculated for each low‐energy isomer (chemical structures in Figure 2 and S1, Supporting Information).

Low‐energy isomer	Substituents	*λ*_abs_[nm, eV]	*f*	*ρ*_s_[kJ g^−1^]
$1_{\text{le}}^{\text{R}}$		307, 4.04	4.86·10^−6^	1.26
$2_{\text{le}}^{1}$	A: ‐NH_2_; B: ‐H	267, 4.64	1.14·10^−6^	1.15
$3_{\text{le}}^{1}$	A: ‐NH_2_; B: ‐NH_2_^+^	288, 4.30	9.48·10^−5^	1.13
$4_{\text{le}}^{1}$	A: ‐CN; B: ‐NH	328, 3.78	3.59·10^−4^	1.07
$5_{\text{le}}^{1}$	A: ‐CN; B: ‐N(Me)	382, 3.25	1.19·10^−4^	0.99
$6_{\text{le}}^{1}$	A: ‐CN; B: ‐N(Et)	380, 3.26	2.58·10^−3^	0.93
$7_{\text{le}}^{1}$	A: ‐CN; B: ‐NH_2_^+^	226, 5.49	4.27·10^−1^	1.07
$8_{\text{le}}^{1}$	A: ‐NH_2_; B: ‐NH	312, 3.98	1.71·10^−6^	1.14
$9_{\text{le}}^{1}$	A: ‐H; B: ‐N(Me)	355, 3.49	1.60·10^−3^	1.15
$10_{\text{le}}^{2}$	A: ‐NH_2_; B: ‐H	266, 4.66	1.14·10^−6^	1.51
$11_{\text{le}}^{2}$	A: ‐NH_2_; B: ‐NH_2_^+^	287, 4.32	9.48·10^−5^	1.49
$12_{\text{le}}^{2}$	A: ‐CN; B: ‐NH	329, 3.77	3.59·10^−4^	1.39
$13_{\text{le}}^{2}$	A: ‐CN; B: ‐N(Me)	381, 3.25	1.19·10^−4^	1.26
$14_{\text{le}}^{2}$	A: ‐CN; B: ‐N(Et)	380, 3.26	2.58·10^−3^	1.15
$15_{\text{le}}^{2}$	A: ‐CN; B: ‐NH_2_^+^	224, 5.54	4.27·10^−1^	1.38
$16_{\text{le}}^{2}$	A: ‐NH_2_; B: ‐NH	311, 3.99	1.71·10^−6^	1.50
$17_{\text{le}}^{2}$	A: ‐H; B: ‐N(Me)	353, 3.51	1.60·10^−3^	1.51
$18_{\text{le}}^{3}$	X: ‐CH; Y: ‐CH	400, 3.10	2.83·10^−6^	2.26
$19_{\text{le}}^{3}$	X: ‐CH; Y: ‐N	527, 2.35	5.76·10^−3^	1.89
$20_{\text{le}}^{3}$	X: ‐N; Y: ‐CH	321, 3.86	1.64·10^−3^	2.35
$21_{\text{le}}^{3}$	X: ‐N; Y: ‐N	550, 2.25	5.76·10^−3^	1.00

The first generation modifies the reference by adding donor and acceptor groups covalently linked to the vinyl moiety (A, B in Figure 2a), following a similar strategy already employed for NBD/QC, here generalized to include all electronically possible push–pull effects (A and B acceptor groups; both donors, acceptors, or one donor and the other acceptor, see Table 1 and Figure S1a, Supporting Information). Depending on the choice of A and B, this can lead to a desired absorption maximum red‐shift (*λ*_abs_ up to 382 nm) thus expecting its absorption tail to enter the visible spectrum, although at the expense of a *ρ*_s_ decrease compared to the reference, similarly to the substituted NBD/QC.

Then, the second generation is meant to avoid this *ρ*_s_ decrease keeping the red‐shift achieved by the first generation, by eliminating the 5‐membered carbon ring originating from the use of cyclopentene during the synthesis.^[^ ^34^ ^]^ Such modification of the first generation compounds does not alter the chromophoric units of the low‐energy compounds, thus maintaining in principle unchanged all photophysical and photochemical properties (see Table 1). Indeed, such a strategy allows a *ρ*_s_ to rise at even higher values than its reference (Figure 2).

Lastly, a third generation was designed aiming at increasing the *π*‐conjugation and, at the same time, avoiding the necessity of donor and acceptor groups. This was accomplished by modifying the alkyl moiety connecting vinyl and carbonyl moieties within the low‐energy isomer through a double bond, thus generating a 6‐π electrons conjugated chromophore. As summarized in Figure 2, this results in a consistent absorption red‐shift, possibly including most of the visible spectrum (*λ*_abs_ up to 550 nm) and maximizing *ρ*_s_ until 2.35 kJ g^−1^.

In the following sections, a detailed explanation of the photophysics and photochemistry processes required to generate the high‐energy isomer for the proposed generations of compounds will be discussed.

### 2.2 UV–Visible Absorption Match with Sunlight Irradiation

**Figure 3:**
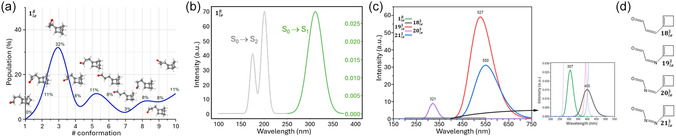
a) Boltzmann distribution of the possible $1_{\text{le}}^{\text{R}}$ ground state conformations (energetics in Figure S2, Supporting Information). b) Simulated $1_{\text{le}}^{\text{R}}$ absorption spectrum, as a weighted convolution from all conformations. c) Simulated absorption spectra of the third‐generation low‐energy species, including d) A comparison with the reference compound.

Due to the flexible alkyl linker connecting both double bonds, the reference low‐energy isomer ($1_{\text{le}}^{\text{R}}$) is expected to populate several conformations in the liquid state. To consider all of them, each torsional conformer $1_{\text{le}}^{\text{R}}$ was identified by mapping its ground state (GS) potential energy surface through both a chemically intuitive‐based and a random search.^[^ ^38^ ^]^ The results are given in **Figure** **3**a in terms of Boltzmann distribution (MP2 Gibbs energies in Figure S2, Supporting Information) showing indeed a complex scenario with four conformations overcoming 10% of the population, with the leading one reaching 32%. All conformations were taken into account to simulate the CASPT2 absorption spectrum as a weighted convolution of Gaussian functions (Figure 3b and S3, Supporting Information), finding a good agreement with the experiment,^[^ ^34^ ^]^ considering both shape and maximum absorption energies of S_0_ → S_1_ and S_0_ → S_2_ bands. Focusing on the lowest in energy S_0_ → S_1_ vertical excitation, (Figure S4 and Table S1, Supporting Information) show how, despite the different spatial arrangement of the alkyl chain, all conformers are characterized by a ^1^(n,π*) excitation, with the n orbital centered on the aldehyde moiety and the π* orbital centered on the vinyl moiety, that is, the two moieties required to come in close contact to form the photoproduct $1_{\text{he}}^{\text{R}}$.

The absorption spectra of first‐ and second‐generation compounds were simulated in the same way, with the S_0_ → S_1_ maximum absorption wavelength shown in Table 1. On one hand, as explained in the previous section, no spectroscopical differences were noted between the two generations, as the removal of the five‐membered ring to obtain the second generation does not affect the chromophoric nature. Therefore, their photophysics and photochemistry are essentially the same. On the other hand, their shifts in absorption compared to the reference are based on the type of substitution pattern (Table 1 and Figure S1a, Supporting Information). Considering the aldehyde moiety as an inherent acceptor moiety, acceptor‐π‐donor push–pull effects were proposed by monosubstituting the A site with the ‐NH_2_ group ($2_{\text{le}}^{1}$, $10_{\text{le}}^{2}$) and by including an additional acceptor group, NH_2_^+^, at the B site ($3_{\text{le}}^{1}$, $11_{\text{le}}^{2}$). In both cases an undesired blue shift suggests attempting the opposite strategy: acceptor group, ‐CN, on the A site and donor group (‐NH, ‐N(Me), ‐N(Et)) on the B site. This way, the higher intramolecular charge transfer character allows a consistent red shift of up to 75 nm (*λ*_abs_ = 382 nm for $5_{\text{le}}^{1}$). Even when monosubstituting the B site with the ‐N(Me) donor group, a firm red shift of 48 nm was found (*λ*_abs_ = 355 nm for $9_{\text{le}}^{1}$). To complete the possible assessment of donor or acceptor groups on A or B sites, acceptor‐π‐acceptor ($7_{\text{le}}^{1}$, $15_{\text{le}}^{2}$) and donor‐π‐donor ($8_{\text{le}}^{1}$, $16_{\text{le}}^{2}$) systems were proposed. The resulting *λ*_abs_ values indicate that, on one hand, acceptor‐π‐acceptor compounds have the same blue‐shift trend as acceptor‐π‐donor compounds, even more pronounced in the presence of an additional acceptor group. On the other hand, donor‐π‐donor compounds show only negligible differences compared to the reference.

The third‐generation compounds are the ones giving rise to the largest bathochromic shift, making it possible to fully cover the visible region. Figure 3c gives a comparison of the S_0_ → S_1_ computed spectra with the reference, and Figure 3d shows the four proposed systems, including a central double bond formed by carbon and/or nitrogen atoms. The success of this design strategy is shown by the extent of the obtained red shift: apart from the limited shift (14 nm) offered by $20_{\text{le}}^{3}$, the other compounds are allowed to cover the blue–green region ($18_{\text{le}}^{3}$) or even the full visible range, 400–750 nm, with absorption maxima centered around 527 nm ($19_{\text{le}}^{3}$) and 550 nm ($21_{\text{le}}^{3}$). From the point of view of the orbitals involved in the electronic transition (Figure S5, Supporting Information), it is confirmed the ^1^(*n*,π*) nature observed for the reference and the previous generations. Actually, a possible drawback of third‐generation compounds compared to the others is studied here, and it is the fact that the corresponding high‐energy isomers still possess an unsaturated double bond and could therefore absorb light, possibly causing an undesired photochemical activation of the generated photoproduct, thus hampering its use as MOST. To clarify this issue, the S_0_ → S_1_ absorption spectra of $18_{\text{he}}^{3}$‐$21_{\text{he}}^{3}$ were simulated and compared with $1_{\text{he}}^{\text{R}}$ as well as with all corresponding low‐energy isomers (Figure S3, Supporting Information). It has to be noted that, in the case of the high‐energy isomer (apart from third generation), two conformations are always present depending on the orientation of the five‐membered carbon ring, as shown (Figure S2, Supporting Information) for $1_{\text{he}}^{\text{R}}$, not differing much in Gibbs energy (0.2–2.7 kcal mol^−1^). The results show that, as supposed, no overlap between low‐energy and high‐energy isomer absorption is expected for the reference system. The same applies to 18 and 19 which can be therefore safely proposed as MOST systems, while especially 20 and partially 21 show a significant overlap, hence compromising in principle their use as MOST.

### 2.3 The Photoinduced Process to Generate Energy‐Storage Species

Although absorption in the visible part of the spectrum is required, it has to be verified that the absorbed photon allows the desired photoproduct formation. Hence, we have studied the CASPT2//CASSCF minimum energy paths following S_0_ → S_1_ absorption for some of the proposed low‐energy isomers. Considering the physicochemical nature of the expected Paternò–Büchi reaction, triplet states were also included, and singlet–triplet population was evaluated by the calculation of the spin‐orbit coupling (SOC) that could allow intersystem crossing.

**Figure 4:**
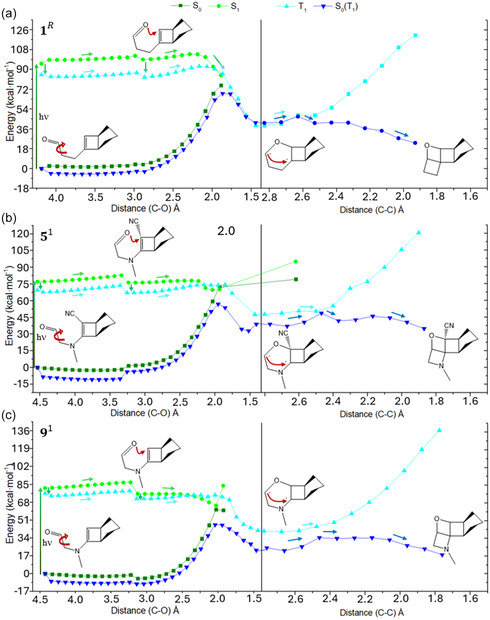
Photoinduced pathways generating the high‐energy isomer (closed form) calculated at CASPT2//CASSCF level of theory, for a) $1_{\text{le}}^{\text{R}}$→$1_{\text{he}}^{\text{R}}$, b) $5_{\text{le}}^{1}$ → $5_{\text{he}}^{1}$, and c) $9_{\text{le}}^{1}$ → $9_{\text{he}}^{1}$. The ground state energy corresponding to the *S*_1_ pathway is referred to as *S*_0_, while the ground state energy corresponding to the *T*_1_ pathway is referred to as *S*_0_(*T*_1_). The arrows on the potential energy surfaces show the possible paths followed by each system after photon absorption, including intermediate structures depicting each photoisomerization step.

Four systems were studied: the reference ($1_{\text{le}}^{\text{R}}$) known to produce by irradiation $1_{\text{he}}^{\text{R}}$ (**Figure** **4**a);^[^ ^34^ ^]^ $5_{\text{le}}^{1}$ as is the most red‐shifted first‐generation compound (Figure 4b); $9_{\text{le}}^{1}$ having the same donor group as $5_{\text{le}}^{1}$ but no acceptor group, allowing to assess the feasibility of generating the C—O σ‐bond due to steric and inductive (‐*I*) effects eventually caused by the —CN group (Figure 4c); $18_{\text{le}}^{3}$ to corroborate the photoisomerization feasibility when including an additional C=C double bond (Figure S6, Supporting Information).

In all cases, the most representative conformation of the low‐energy isomer was taken as Franck–Condon structure. For the reference and the first‐generation systems (Figure 4), this corresponds to an extended alkyl linker requiring, after photon absorption, rotation along a single C—C bond to bring closer the oxygen atom to the carbon of the vinyl moiety (from ≈4.2 to 2.8 Å). This can be obtained by overcoming an *S*_1_ energy barrier of 5–6 kcal mol^−1^ or after *S*_1_ → *T*_1_ population (SOC at Franck–Condon structure: 1.2–1.4 cm^−1^) through a slightly lower *T*_1_ barrier of 4–5 kcal mol^−1^. After rotation, the *S*_1_ → *T*_1_ SOC increases to 4.8–5.2 cm^−1^. From the *S*_1_ or *T*_1_ rotated minima, the C—O bond formation requires additional energy especially for $1^{\text{R}}$ (5 and 7 kcal mol^−1^ in *S*_1_ and *T*_1_, respectively), while an almost barrierless (2 kcal mol^−1^) and completely barrierless reaction is expected for $5^{1}$ and $9^{1}$, respectively, in *S*_1_. This lets us conclude that the presence of an acceptor group linked to the reactive carbon does not hamper the bond formation, while the inclusion of a nitrogen atom in the alkyl chain increases its feasibility. This is most probably due to the higher flexibility that a nitrogen atom confers to the alkyl chain compared to a fully carbonated chain, since bending (inversion) complements torsion.^[^ ^39^ ^]^ In all three systems, the *S*_1_‐driven C—O bond formation is possible through an *S*_1_/*S*_0_ conical intersection or directly on the *T*_1_ surface. In both cases, the result is a diradical compound leading to two possible pathways: C—C bond formation directly in *S*_0_ or after *T*_1_ → *S*_0_ intersystem crossing. The former pathway requires energy activation, although in all cases the excess energy given by the energy difference between *S*_1_/*S*_0_ conical intersection and *S*_0_ local minimum (corresponding to the diradical) is enough to overcome such a barrier. The latter pathway could be even preferred for the first‐generation systems, since it would result in an almost energy‐barrierless process.

All in all, we can conclude that the proposed substitution patterns favor C—O bond formation in both *S*_1_ and *T*_1_ states compared to the reference, with an expected highly feasible production of the high‐energy isomer through an only‐singlet *S*_0_ → *S*_1_ → *S*_0_ pathway or a singlet‐triplet‐singlet *S*_0_ → *S*_1_ → *T*_1_ → *S*_0_ pathway. *S*_1_ → *T*_1_ hopping is possible at different reaction stages and with different probability, as suggested by the non‐negligible SOC values.

The different chromophore nature of third‐generation $18_{\text{le}}^{3}$ leads to a slightly different photochemistry (Figure S6, Supporting Information). After *S*_0_ → *S*_1_ photon absorption, two lower lying triplet states are in principle available, although *T*_1_ should be populated instead of *T*_2_, due to a consistently higher SOC (11.3 compared to 0.07 cm^−1^) as expected following the El‐Sayed's rule, since both *S*_1_ and *T*_2_ are (*n*,π*) states.^[^ ^40^ ^]^ Nonetheless, C—O bond formation can occur in both *S*_1_ and *T*_1_ states by overcoming a ≈10 kcal mol^−1^ energy barrier, although the excess energy given by the absorbed photon favors the triplet manifold mechanism. Moreover, differently from reference and first‐generation systems, the *S*_1_/*S*_0_ conical intersection is not observed, hence clarifying that the *S*_0_ → *S*_1_ → *T*_1_ → *S*_0_ pathway is the only available pathway. Indeed, after reaching a *T*_1_ minimum corresponding to the diradical intermediate, a high‐lying *T*_1_/*S*_0_ crossing (≈20 kcal mol^−1^) allows population of the closed‐shell GS (SOC: 11.3 cm^−1^), through which the C—C bond is formed corresponding to $18_{\text{he}}^{3}$.

Hence, the conjugated linker between carbonyl and vinyl moieties offering the most suitable absorption properties result, at the same time, in a stiffer linker decreasing the feasibility of the high‐energy photoproduction yet not hampering it.

It has to be mentioned that, from the perspective of a technological application, possible side reactions should be checked due to the presence of an aldehyde group in all proposed systems, which is inherently highly reactive and sensitive to pH. It is advisable to operate at neutral pH to prevent pH‐driven side reactions, such as aldol reactions, and to stabilize the keto form relative to the enol or enolate forms. Furthermore, it is known that the Paternò–Büchi reaction can compete with other pathways depending on experimental conditions. For example, the Norrish Type II reaction involving *γ*‐hydrogen abstraction (especially relevant with electron‐poor alkenes)^[^ ^41^, ^42^ ^]^ or intermolecular Paternò–Büchi reactions, which are concentration‐dependent,^[^ ^42^, ^43^ ^]^ may reduce the quantum yield of the targeted MOST reactivity (i.e., intramolecular Paternò–Büchi). Nevertheless, for the proposed $1_{\text{le}}^{\text{R}}$ system, a reasonably high quantum yield of 55% has been experimentally found, not reporting any secondary products.^[^ ^34^ ^]^

### 2.4 Releasing the Stored Energy as Heat: The Thermal Pathways

Once the high‐energy isomer is generated after photon absorption, it is fundamental to study its thermal stability and its thermal pathway to reverse the system back toward the initial low‐energy isomer, since such information is directly related to the estimated lifetime of the high‐energy isomer (i.e., the MOST storage time) and to the feasibility of the energy‐releasing step.

**Figure 5:**
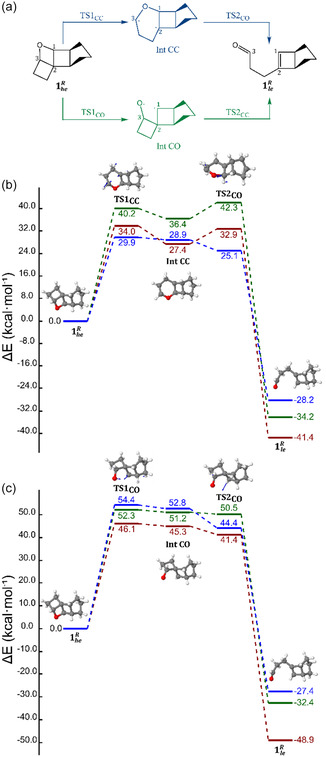
a) Scheme depicting the two possible $1_{\text{he}}^{\text{R}}$ → $1_{\text{le}}^{\text{R}}$ thermal routes to release the stored chemical energy: breaking first the C—C (blue pathway) or the C—O (green pathway) *σ*‐bond, including the formation of the corresponding diradical intermediate. b,c) The energetics of the two routes, respectively, including the structures of each saddle point (the transition state arrows indicate the direction dictated by the imaginary frequency). The energy profiles are given at CASSCF/6‐31G(d) (red), CASSCF/ANO‐S//CASSCF/6‐31G(d) (green), and CASPT2/ANO‐S//CASSCF/6‐31G(d) (blue) level of theory.

Considering the systems studied here, we can hypothesize two possible two‐step thermal reactions: C—C bond breaking followed by C—O bond breaking, or the opposite (see **Figure** **5**a). In both cases, rupture of the first *σ*‐bond should result in the formation of a highly energetic intermediate, due to its diradical character and therefore, it is expected that the bottleneck of the thermal reversion process is constituted by the first energy barrier. In other words, the high‐energy isomer will revert following the path requiring the lowest energy barrier among breaking first the C—C bond or the C—O bond. The results for the $1_{\text{he}}^{\text{R}}$ → $1_{\text{le}}^{\text{R}}$ thermal routes can be found in Figure 5b,c. The three levels of theory (see the Methods section for details) do show that C—C bond breaking happens first, followed by the formation of a six‐membered ring diradical rapidly evolving toward C—O bond breaking. Focusing on the highest level of theory among the three (CASPT2/ANO‐S//CASSCF/6‐31G(d), in blue), the barrier to initially produce a C—C break is 29.9 kcal mol^−1^ (Figure 5b) against the 54.4 kcal mol^−1^ necessary to break first the C—O bond (Figure 5c). Moreover, a comparison of CASPT2 with the other two CASSCF levels of theory (in green and red) highlights the consistent stabilization of the C—C bond‐breaking transition state (TS1_CC_) due to the inclusion of the dynamic electron correlation, contrary to the C—O bond‐breaking transition state (TS1_CO_) destabilization.

The same treatment was performed for $5_{\text{he}}^{1}$ → $5_{\text{le}}^{1}$ (Figure S7b,c, Supporting Information) and $9_{\text{he}}^{1}$ → $9_{\text{le}}^{1}$ (Figure S7d,e, Supporting Information) thermal routes, that is, the two first‐generation systems, in which photochemistry was studied in detail in the previous section. For the two systems, it is confirmed the same trend that was observed for the reference. The C—C bond is expected to break first followed straightforwardly by the C—O bond. Looking at the different theoretical treatments, we observe again how the dynamic electron correlation (CASPT2 results) tends to stabilize more TS1_CC_ than TS1_CO_. Especially, lower energy barriers are expected compared to reference. An energy barrier of 18.7 and 17.2 kcal mol^−1^ is required to break first the C—C bond in $5_{\text{he}}^{1}$ and $9_{\text{he}}^{1}$, respectively, while 44.0 and 51.2 kcal mol^−1^ are needed to break first their C—O bond.

**Figure 6:**
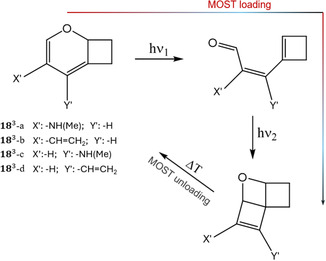
MOST mechanism of third‐generation compounds, including all studied substituents for the $18^{3}$system.

A different scenario is observed for the third‐generation compounds $18_{\text{he}}^{3}$ (Figure S8b,c, Supporting Information) and $21_{\text{he}}^{3}$ (Figure S8d,e, Supporting Information), the former including an additional C=C bond and the latter an additional N=N bond (see Figure 2). Also, for these systems breaking first the C—C bond is preferred, with energy barriers comparable to reference: 25–26 kcal mol^−1^ for reaching TS1_CC_ versus 59–60 kcal mol^−1^ for reaching TS1_CO_ at CASPT2//CASSCF level. Nevertheless, the preferred path leads to an “intermediate“ (Int CC) more stable than the low‐energy isomer, as confirmed by CASPT2 fully optimized le and Int CC structures (Table S2, Supporting Information). From the chemical point of view, this is due to the fact that, in third‐generation compounds, C—C breaking does not result in an unstable diradical but in a stable bicycle compound. As a matter of fact, this modifies completely the expected MOST functioning mechanism, since the low‐energy isomer corresponds to the initially expected Int CC structure. We have therefore reconsidered the photoinduced pathway for the $18^{3}$ system, starting from this bicycle structure and including the substituent effect by introducing a donor group, —NH(Me) or vinyl, in all its possible orientations (**Figure** **6**). The newly established low‐energy isomer absorbs a photon, breaking in an ultrafast fashion (through an energy barrierless pathway leading to an *S*_1_/*S*_0_ conical intersection) the C—O bond, and thus forming the extended conformation previously considered as a low‐energy isomer. The irradiation of such extended conformation was already described in the previous section (Figure S6, Supporting Information), and it results in the formation of the high‐energy isomer. From there, thermal C—C breaking brings back the bicyclic low‐energy isomer. All in all, third‐generation compounds can therefore work as MOST systems, but necessitating the sequential absorption of two photons in order to store energy. When appropriately substituted (vinyl in *para* position to the oxygen, $18^{3}$‐d) both photons’ energy falls in the visible range (402 and 412 nm, Table S3, Supporting Information) and the resulting high‐energy isomer is, as desired, photostable in the visible and near‐UV regions (values in Table S3, Supporting Information for the $18^{3}$‐a–d series). The drawback is the *ρ*_s_ value, decreasing to 0.90 kJ g^−1^ as a consequence of the increased molar mass due to substitution.

### 2.5 Bioinspired Energy Release: The Enzymatic Activity

As discussed in the previous section, apart from third‐generation compounds, a retro‐Paternò–Büchi reaction is necessary to release the stored energy and recover the initial low‐energy isomer. Moreover, in all cases, the thermal pathway requires a C—C bond breaking. Although a retro‐Paternò–Büchi reaction is uncommon, it was proposed by electrochemical methods, indeed obtaining the successive homolytic cleavage of C—C and C—O bonds.^[^ ^44^ ^]^

**Figure 7:**
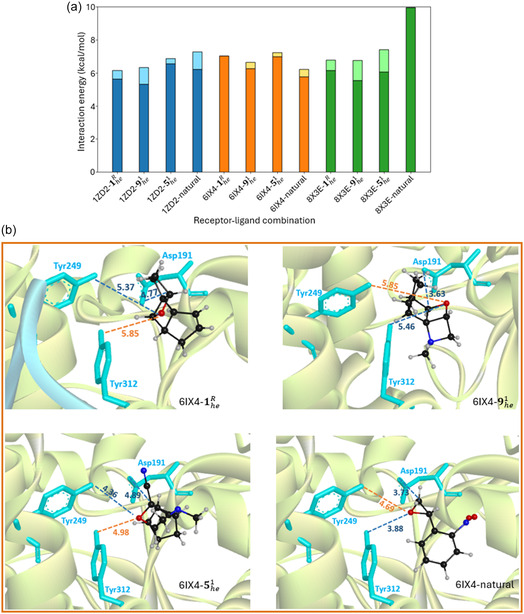
a) Interaction energy for different receptor–ligand combinations, including lower and upper boundaries (darker and lighter colors, respectively). The results refer to the receptors: human epoxide hydrolase (blue), *Aspergillus usamii* epoxide hydrolase (orange), *Taxus cuspidata* taxadiene‐5α‐hydroxylase (green), defined by their PDB codes. b) The position and orientation of representative poses, corresponding to the highest interaction energies for *Aspergillus usamii* epoxide hydrolase, are shown, including the most relevant distances, in Ångström, between the ligand and the Asp191‐Tyr249‐Tyr312 catalytic triad (the most distant side chain is indicated in orange). See (Figure S10, Supporting Information) for the remaining receptor‐ligand structures.

Here, we propose the use of enzymatic reactivity to compensate for the almost complete absence of known chemical reactions favoring a retro‐Paternò–Büchi mechanism. More in detail, we looked for enzymes known to have an active site catalyzing this type or similar reactions, which X‐ray structure is available to perform a docking study. This way, it was screened the probability of formation of the ligand‐enzyme complex which, if eventually stable, could ensure a retro‐Paternò–Büchi reaction. Eight enzymes were selected^[^ ^45^, ^46^, ^47^, ^48^, ^49^, ^50^, ^51^, ^52^, ^53^ ^]^ (see Table S4, Supporting Information) and four ligands were considered: the reference, first‐generation $5_{\text{he}}^{1}$ and $9_{\text{he}}^{1}$, and the respective natural ligand, used as a positive control. The highest interaction energies are given as a heatmap in (Figure S9, Supporting Information), showing how three enzymes outperform: human epoxide hydrolase (PDB code: 1ZD2),^[^ ^46^ ^]^ *Aspergillus usamii* epoxide hydrolase (6IX4),^[^ ^45^ ^]^ and *Taxus cuspidata* taxadiene‐5α‐hydroxylase (8X3E).^[^ ^47^, ^48^ ^]^ Lower and upper boundaries of their interaction energy are given in **Figure** **7**a, showing in all cases a ≈5–7 kcal mol^−1^ interaction energy, competing and in some cases exceeding the interaction energy of the natural ligand‐enzyme complex, apart from *Taxus cuspidata* taxadiene‐5α‐hydroxylase.

Since a key factor in enzymatic reactivity, apart from the interaction energy with the ligand, is the orientation of the ligand within the active site, we have identified and analyzed representative docking poses. Concerning *Aspergillus usamii* epoxide hydrolase (Figure 7b), it is remarkable how the size of the natural ligand, *o*‐nitrostyrene oxide, is similar to the proposed high‐energy MOST systems. Moreover, there is a match in their position with respect to the Asp191‐Tyr249‐Tyr312 catalytic triad. Especially, $1_{\text{he}}^{\text{R}}$ and $5_{\text{he}}^{1}$ also show the same orientation with respect to the oxygen atom. Indeed, for this enzyme, the interaction energy of all the proposed MOST systems is slightly higher than the natural ligand. Concerning the catalytic mechanism, it is known that it starts with a proton transfer from either Tyr249 or Tyr312 side chain toward the ligand.^[^ ^45^ ^]^ In this respect, $5_{\text{he}}^{1}$ shows the shortest distances compared to the other MOST ligands, with the distance to Tyr249 in very good agreement compared to the natural ligand (Figure 7b).

In the cases of human epoxide hydrolase and *Taxus cuspidata* taxadiene‐5α‐hydroxylase (Figure S10a,b, Supporting Information), the MOST ligands can also be accommodated in the active site. Nevertheless, their lower size compared to the respective natural ligands is evidenced by their capability to interact at different sides of the same active site, making in principle less specific the interaction. Indeed, apart from $5_{\text{he}}^{1}$ interacting with human epoxide hydrolase, the MOST ligands show a lower interaction energy with these two receptors, compared with the natural ligand. Hence, we do expect *Aspergillus usamii* epoxide hydrolase to be the candidate with higher probability to efficiently catalyze energy release of the proposed MOST systems. This is particularly intriguing since *Aspergillus usamii*, like other species of the *Aspergillus* genus, is known for its metabolic versatility and suitability for enzyme engineering,^[^ ^54^, ^55^ ^]^ making its epoxide hydrolase a promising starting point for the optimization of the catalytic activity required to release the energy stored by the proposed MOST systems.

## 3 Conclusion

We have proposed, through the application of different levels of theory spanning from molecular docking to ab initio multiconfigurational quantum chemistry, a novel class of compounds inspired by experimental results offering a potential breakthrough in MOST technology. Starting from an initial experimentally conceivable compound, three different generations were designed based on the optimization of all parameters that could be controlled by computational approaches: absorption spectrum, storage density, and a detailed description of MOST loading and unloading paths including eventual drawbacks. Moreover, to the best of our knowledge, enzymatic catalysis was proposed for the first time as sustainable and innovative way to release the stored energy, by proposing enzymatic active sites based on the enzyme function and specific reactivity.

All in all, we have found that the Paternò–Büchi reaction can potentially serve as photoreaction to generate highly strained high‐energy oxetanes offering the possibility to store a large amount of energy compared to the known norbornadiene/quadricyclane couple, until now considered as reference for MOST applications. If properly substituted, the proposed low‐energy compounds can cover most of the visible absorption spectrum. Photon absorption is followed by excited state (ES) C—O and GS C—C bond formation, responsible for the generation of the oxetane ring. Hence, this study offers also mechanistic details on the required interplay between singlet and triplet manifolds of an intramolecular Paternò–Büchi reaction.

The necessary retro‐Paternò–Büchi reaction to open the oxetane ring forming back the low‐energy isomer, mainly unknown through any kind of experimental chemical method, is proposed through known enzymatic activity. In the future, apart from encouraging the extension of experimental studies of such compounds, we envision widening the computational study concerning the underneath enzymatically induced mechanisms.

## 4 Experimental Section

4.1

4.1.1

##### Quantum Chemistry Calculations

All low‐energy and high‐energy structures were fully optimized at MP2/6‐31G(d) level of theory through the Gaussian16 software.^[^ ^56^ ^]^ Frequency calculations confirmed the minimum nature of the optimized geometry. The Torsiflex program^[^ ^38^ ^]^ was used to perform an exhaustive conformational search of the low‐energy isomers. All absorption spectra and ES minimum energy paths were calculated by multiconfigurational CASPT2//CASSCF methodology, that is, CASPT2^[^ ^57^ ^]^ energy corrections on top of CASSCF^[^ ^58^ ^]^ optimized structures. To test the validity of this approach, full CASPT2 optimization of selected compounds corresponding to specific energy minima was performed, indicating a negligible energy difference of maximum 0.06 kcal mol^−1^ between CASPT2//CASSCF and CASPT2 approaches (Table S2, Supporting Information). More in detail, state‐averaged (SA‐)CASSCF^[^ ^59^ ^]^ and multistate (MS‐)CASPT2^[^ ^60^ ^]^ calculations were performed, including five singlet and five triplet states. In CASPT2 calculations, an imaginary shift of 0.2 was included to avoid intruder states. No IPEA shift was included to avoid any bias.^[^ ^61^ ^]^ All thermal pathways were calculated by identifying saddle points (minima and transition states) at CASSCF/6‐31G(d) level of theory. The effect of the basis set was considered by single‐point calculations at CASSCF/ANO‐S level, while the effect of including the dynamic electron correlation was considered by single‐point calculations at CASPT2/ANO‐S level. All photoinduced pathways were calculated at CASPT2/ANO‐S//CASSCF/6‐31G level.

This balanced solution was applied as a result of a benchmark of different active spaces and different basis sets on the four lowest‐energy singlet ES of the most stable conformation of the reference $1_{\text{le}}^{\text{R}}$ (Figure S4b, Supporting Information). On the basis of this same benchmark, an active space of 8 electrons in 6 orbitals (8,6) was chosen for $1_{\text{le}}^{\text{R}}$, allowing to select all *π* and π* orbitals, and the two *n* orbitals on the oxygen atom. The same (8,6) active site was also applied to first‐and second‐generation systems in order to have a direct comparison with the reference, after having checked the effect of larger active spaces. Third‐generation compounds required a larger active space, due to the increased *π*‐conjugation: (10,8) for $18_{\text{le}}^{3}$, (12,9) for $19_{\text{le}}^{3}$ and $20_{\text{le}}^{3}$, (14,10) for $21_{\text{le}}^{3}$, that is, including all *π*, π*, and n orbitals.

A conformational analysis was performed on low‐energy and high‐energy species, with the exception of high‐energy third‐generation compounds possessing a single possible conformation (see Figure S2, Supporting Information for $1_{\text{le}}^{\text{R}}$ and $1_{\text{he}}^{\text{R}}$). The enthalpy difference (ΔH) necessary for obtaining the energy‐storage density (*ρ*_s_) of each MOST system was calculated referring to the high‐energy conformer and the low‐energy conformer showing, among all possible conformers, the lowest Gibbs energy, hence corresponding to the conformers with the highest population within their respective Boltzmann distribution.

All CASSCF and CASPT2 calculations were performed with the OpenMolcas suite of programs.^[^ ^62^ ^]^ Molecular orbitals were visualized with the Jmol viewer.^[^ ^63^ ^]^

##### Molecular Docking Calculations

The geometry of the three oxetane ligands ($1_{\text{he}}^{\text{R}}$,$5_{\text{he}}^{1}$, $9_{\text{he}}^{1}$) was fully optimized at MP2/6‐31G(d) level of theory as indicated in the previous section. Each natural ligand of its respective enzyme was downloaded from the PDB and prepared by assigning Gasteiger charges as partial atomic charges and by defining torsional degrees of freedom, via AutoDockTools.^[^ ^64^ ^]^ For each receptor (i.e., enzyme) the same procedure concerning structure download from PDB and Gasteiger charges assignment was followed. Targeted docking was then performed by selecting a box centered on the known active site (see Table S5, Supporting Information for position and size of the box). Molecular docking was then performed with the AutoDock Vina software,^[^ ^65^, ^66^ ^]^ running 50 independent calculations each generating 40 ligand–receptor poses, resulting in a total of 900 poses per ligand–receptor couple. The results were visually inspected with BIOVIA Discovery Studio Visualizer.^[^ ^67^ ^]^

## Conflict of Interest

The author declare no conflicts of interest.

## Supporting information

Supplementary Material

## Data Availability

The data that support the findings of this study are available in the supplementary material of this article.
