# Supplementary material for: Designing Molecular Solar Thermal Systems Based on the Paternò–Büchi Reaction Coupled to Enzymatic Energy Release
Source: ChemSusChem. 2025 Jun 16;18(15):e202500777. doi: 10.1002/cssc.202500777 (PMC12302321; doi:10.1002/cssc.202500777)
Supplement: Supplementary file 1 — Supplementary Material [file CSSC-18-e202500777-s001.pdf]

ChemSusChem

Supporting Information

Designing Molecular Solar-Thermal Systems Based on the Paternò-Büchi Reaction Coupled to Enzymatic Energy Release

Marta Delgado-Gómez,[a] Jesús Reategui Illatopa,[a] Lorenzo Gramolini,⊥[a] Richard López Corbalán,⊥[a] Cristina García-Iriepa,[a,b] and Marco Marazzi*[a,b]

[a] Universidad de Alcalá, Departamento de Química Analítica, Química Física e Ingeniería Química, Functional Molecular Systems (FuMSys) group
Ctra. Madrid-Barcelona, km. 33,600, 28805, Alcalá de Henares (Madrid), Spain
E-mail: marco.marazzi@uah.es

[b] Universidad de Alcalá, Instituto de Investigación Química "Andrés M. del Río"
Ctra. Madrid-Barcelona, km. 33,600, 28805, Alcalá de Henares (Madrid), Spain

⊥ These authors contributed equally.

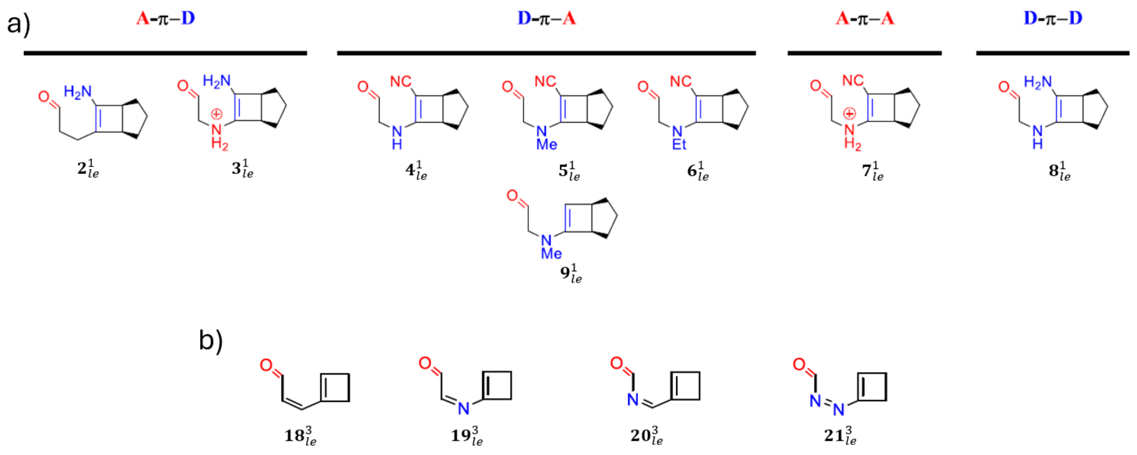

Figure S1. Low-energy isomers of a) 1st generation and b) 3rd generation. The 2nd generation is equivalent to the 1st one, after eliminating the 5-membered carbon ring, thus decreasing the corresponding molecular weight. Donor and acceptor substituents/moieties are shown in blue and red, respectively.

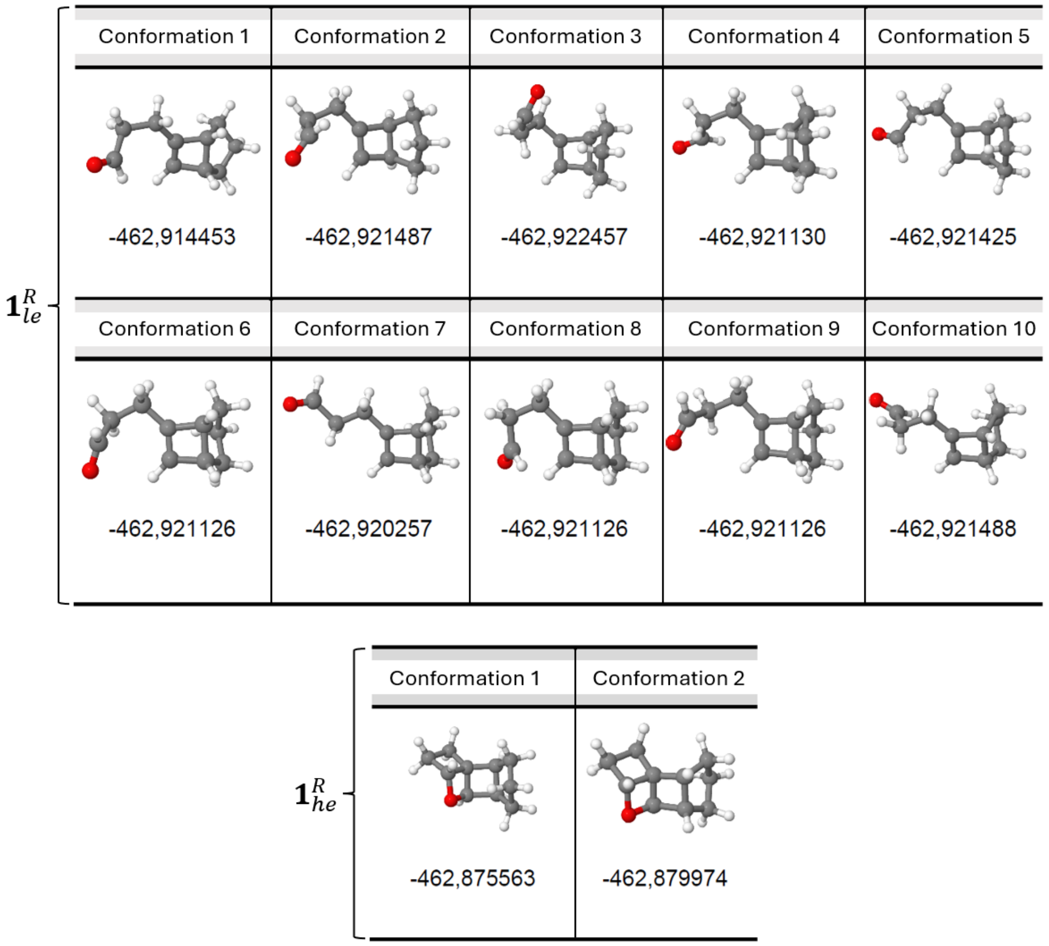

Figure S2. MP2/6-31G(d) optimized geometries (i.e., local minima on the ground state potential energy surface) of each $\mathbf{1}_{le}^{R}$ and $\mathbf{1}_{he}^{R}$ conformer, including its Gibbs free energy (G) in Hartree.

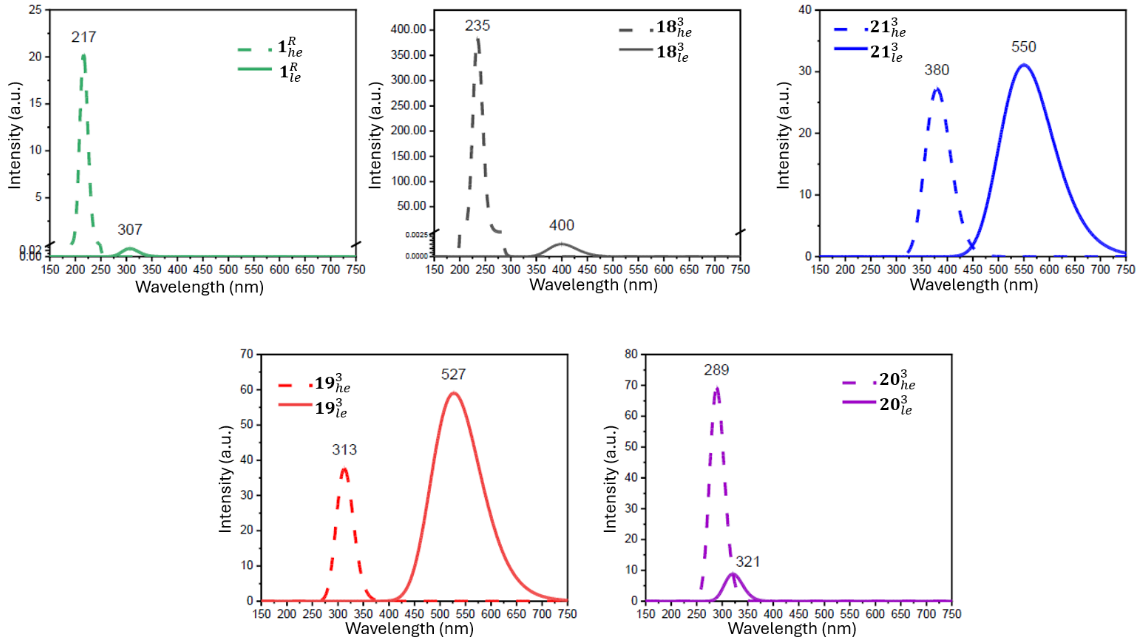

Figure S3. Absorption spectra covering the full UV-visible range (150-750 nm) of the reference and of the proposed 3rd generation MOST systems, including low-energy (*le*) and high-energy (*he*) isomers.

a)

Orbital	$\mathbf{1}_{le}^{R}$ conformation 3 Representation	$\mathbf{1}_{le}^{R}$ conformation 3 Type	$\mathbf{1}_{le}^{R}$ conformation 10 Representation	$\mathbf{1}_{le}^{R}$ conformation 10 Type
**43** **LUMO +1**	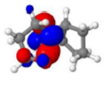	π*(C=C) + π*(C=O)	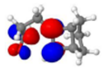	π*(C=O) + π*(C=C)
**42** **LUMO**	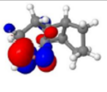	π*(C=O) + π*(C=C)	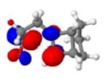	π*(C=O) + π*(C=C)
**41** **HOMO**	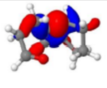	σ + n	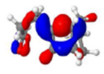	σ*
**40** **HOMO -1**	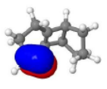	π(C=O)	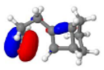	π(C=O)
**39** **HOMO -2**	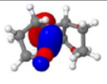	π(C=C) + n	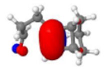	π(C=C) + n
**38** **HOMO -3**	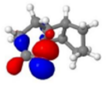	n	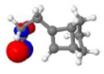	n

b)

PES transition	Active space	6-31G* Transition energy (eV)	6-31G* Oscillator strength *f*	ANO-S Transition energy (eV)	ANO-S Oscillator strength *f*	ANO-L Transition energy (eV)	ANO-L Oscillator strength *f*
**$S_0 \rightarrow S_1$**	**6,5**	6,65	4,029E-01	6,14	1,667E-02	6,25	1,442E-02
	**8,6**	4,19	1,432E-06	4,04	4,860E-06	4,02	4,897E-06
	**10,8**	4,19	1,482E-06	4,19	2,632E-05	4,04	2,583E-05
**$S_0 \rightarrow S_2$**	**6,5**	6,94	3,667E-02	6,55	1,136E-02	6,66	9,120E-03
	**8,6**	7,62	9,060E-03	7,14	7,718E-03	7,08	7,487E-03
	**10,8**	7,70	1,003E-02	6,08	3,255E-01	6,03	3,242E-01
**$S_0 \rightarrow S_3$**	**6,5**	7,57	1,710E-02	7,29	8,355E-03	7,39	8,173E-03
	**8,6**	8,09	2,146E-05	7,79	4,881E-05	7,78	5,359E-05
	**10,8**	8,09	2,516E-05	7,05	8,172E-03	7,00	8,013E-03
**$S_0 \rightarrow S_4$**	**6,5**	7,97	1,553E-02	7,58	1,503E-02	7,68	1,494E-02
	**8,6**	10,17	5,852E-04	9,91	1,406E-03	9,91	1,520E-03
	10,8	*10,16*	*6,801E-04*	*7,64*	*8,325E-03*	*7,65*	*7,952E-03*

Figure S4. a) CASSCF molecular orbitals of the two most relevant ground state $\mathbf{1}_{le}^{R}$ conformations, in terms of population of the Boltzmann distribution (see Figure 3a of the main text). b) Effect of the active space (n electrons, m orbitals) and of the basis set on the four lowest-energy singlet electronic transitions of $\mathbf{1}_{le}^{R}$ (conformation 3) both in terms of transition energy and oscillator strength.

Table S1. CASPT2 $S_0 \rightarrow S_1$ energies for all secondary conformations of $\mathbf{1}_{le}^{R}$, i.e., all conformations but 3.

$\mathbf{1}_{le}^{R}$ conformation	$S_0 \rightarrow S_1$ energy eV	$S_0 \rightarrow S_1$ energy nm	*f*	Transition
1	3,98	311,54	3,093E-06	H_{-3}^{38} (**n**) → L^{42} (**π*cc + π*co**)
2	4,04	306,76	4,860E-06	H_{-3}^{38} (**n**) → L^{42} (**π*co**)
4	3,98	311,36	2,507E-06	H_{-3}^{38} (**n**) → L^{42} (**π*cc + π*co**)
5	4,04	307,11	8,123E-06	H_{-3}^{38} (**n**) → L^{42} (**π*co**)
6	3,98	311,28	2,495E-06	H_{-3}^{38} (**n**) → L^{42} (**π*cc + π*co**)
7	3,99	310,93	4,379E-05	H_{-3}^{38} (**n**) → L^{42} (**π*co**)
8	3,98	311,28	2,494E-06	H_{-3}^{38} (**n**) → L^{42} (**π*cc + π*co**)
9	3,98	311,29	2,478E-06	H_{-3}^{38} (**n**) → L^{42} (**π*cc + π*co**)
10	3,98	311,52	3,103E-06	H_{-3}^{38} (**n**) → L^{42} (**π*cc + π*co**)

$\mathbf{18}_{le}^{3}$

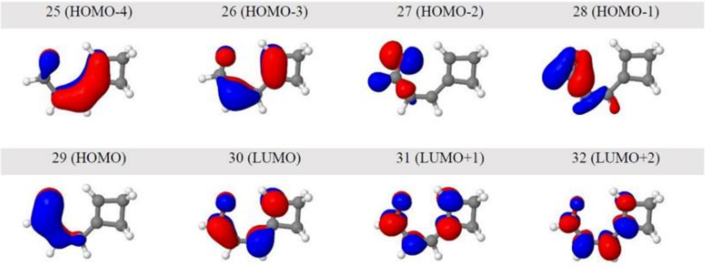

PES transition	Electronic transition	Involved molecular orbitals
$S_0 \rightarrow S_1$	22222000 → 22u22d00	$H_{-2}^{27}(\mathbf{n}_{O}) \rightarrow L^{30}(\boldsymbol{\pi}^*_{CO,CC1,CC2})$
$S_1 \rightarrow T_1$	22u22d00 → 2222u00u	$H^{29}(\boldsymbol{\pi}_{CO,CC}) \rightarrow L_{+2}^{31}(\boldsymbol{\pi}^*_{CO,CC1,CC2})$<br>$L^{30}(\boldsymbol{\pi}^*_{CO,CC1,CC2}) \rightarrow H_{-2}^{27}(\mathbf{n}_{O})$
$S_1 \rightarrow T_2$	22u22d00 → 222u200u	$H_{-1}^{28}(\boldsymbol{\pi}_{CO}) \rightarrow L_{+2}^{31}(\boldsymbol{\pi}^*_{CO,CC1,CC2})$<br>$L^{30}(\boldsymbol{\pi}^*_{CO,CC1,CC2}) \rightarrow H_{-2}^{27}(\mathbf{n}_{O})$
$T_2 \rightarrow T_1$	222u200u → 2222u00u	$H^{29}(\boldsymbol{\pi}_{CO,CC}) \rightarrow H_{-1}^{28}(\boldsymbol{\pi}_{CO})$
$T_1 \rightarrow S_0$	2222u00u → 22222000	$L_{+2}^{32}(\boldsymbol{\pi}^*_{CO,CC1,CC2}) \rightarrow H^{29}(\boldsymbol{\pi}_{CO,CC})$

$\mathbf{21}_{le}^{3}$

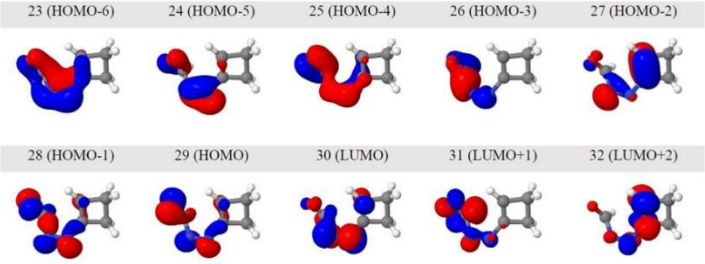

PES transition	Electronic transition	Involved molecular orbitals
$S_0 \rightarrow S_1$	2222222000 → 222222ud00	$H^{29}(\mathbf{n}_{O,N1,N2}) \rightarrow L^{30}(\boldsymbol{\pi}^*_{CO,NN,CC})$
$S_1 \rightarrow T_1$	222222ud00 → 222222u00u	$L^{30}(\boldsymbol{\pi}^*_{CO,NN,CC}) \rightarrow L_{+2}^{32}(\boldsymbol{\pi}^*_{CO,NN})$
$S_1 \rightarrow T_2$	222222ud00 → 22222u200u	$L^{30}(\boldsymbol{\pi}^*_{CO,NN,CC}) \rightarrow H^{29}(\mathbf{n}_{O,N1,N2})$<br>$H_{-1}^{28}(\mathbf{n}_{O,N1,N2}) \rightarrow L_{+2}^{32}(\boldsymbol{\pi}^*_{CO,NN})$
$T_2 \rightarrow T_1$	22222u200u → 222222u00u	$H^{29}(\mathbf{n}_{O,N1,N2}) \rightarrow H_{-1}^{28}(\mathbf{n}_{O,N1,N2})$
$T_1 \rightarrow S_0$	222222u00u → 2222222000	$L_{+2}^{32}(\boldsymbol{\pi}^*_{NN,CC}) \rightarrow H^{29}(\mathbf{n}_{O,N1,N2})$

Figure S5. CASSCF molecular orbitals (left) and corresponding electronic transitions (right) among singlet (S) and triplet (T) states (PES: Potential Energy Surface) relevant to describe the absorption and photochemistry of the 3rd generation compounds $\mathbf{18}_{le}^{3}$ (up) and $\mathbf{21}_{le}^{3}$ (bottom). Active spaces: (10,8) for $\mathbf{18}_{le}^{3}$; (14,10) for $\mathbf{21}_{le}^{3}$.

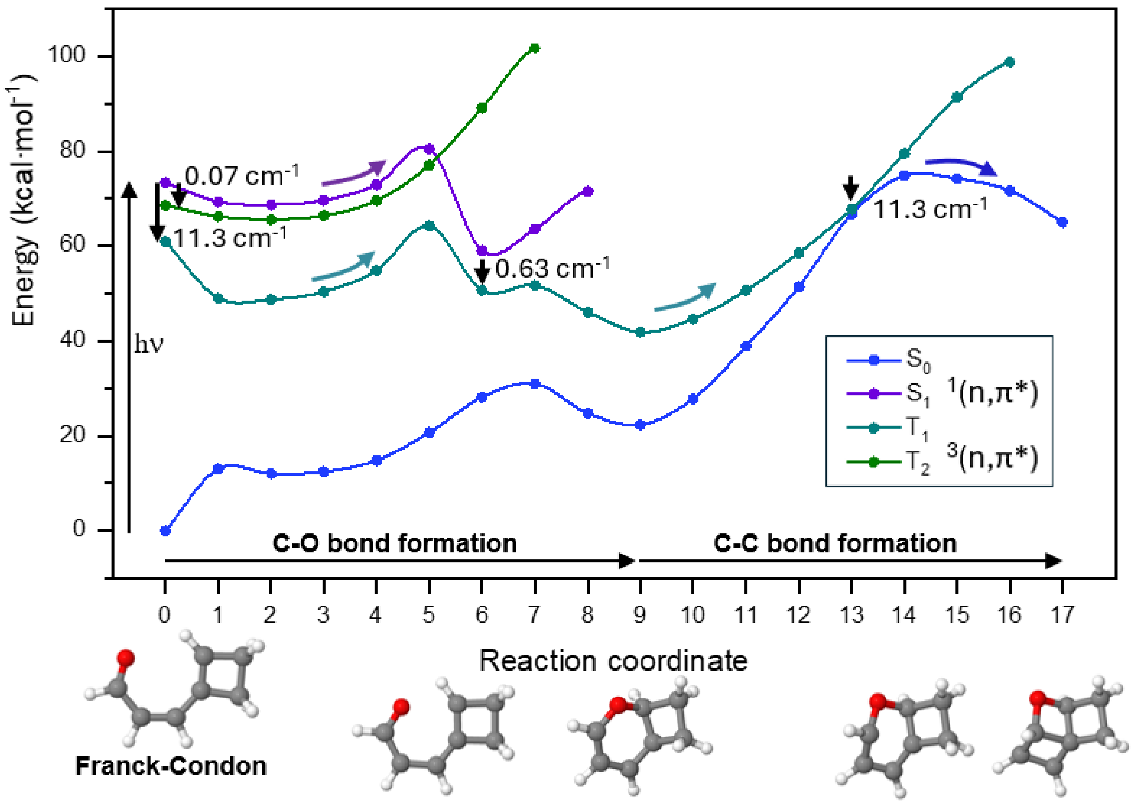

Figure S6. Photoinduced pathways generating the high-energy isomer (closed form) calculated at CASPT2//CASSCF level of theory, for $\mathbf{18}^3_{le} \rightarrow \mathbf{18}^3_{he}$. The arrows on the potential energy surfaces show the possible paths followed by each system after photon absorption, including intermediate structures depicting each photoisomerization step. Spin-orbit coupling (SOC) values are given in cm^{-1} for critical points along the pathway.

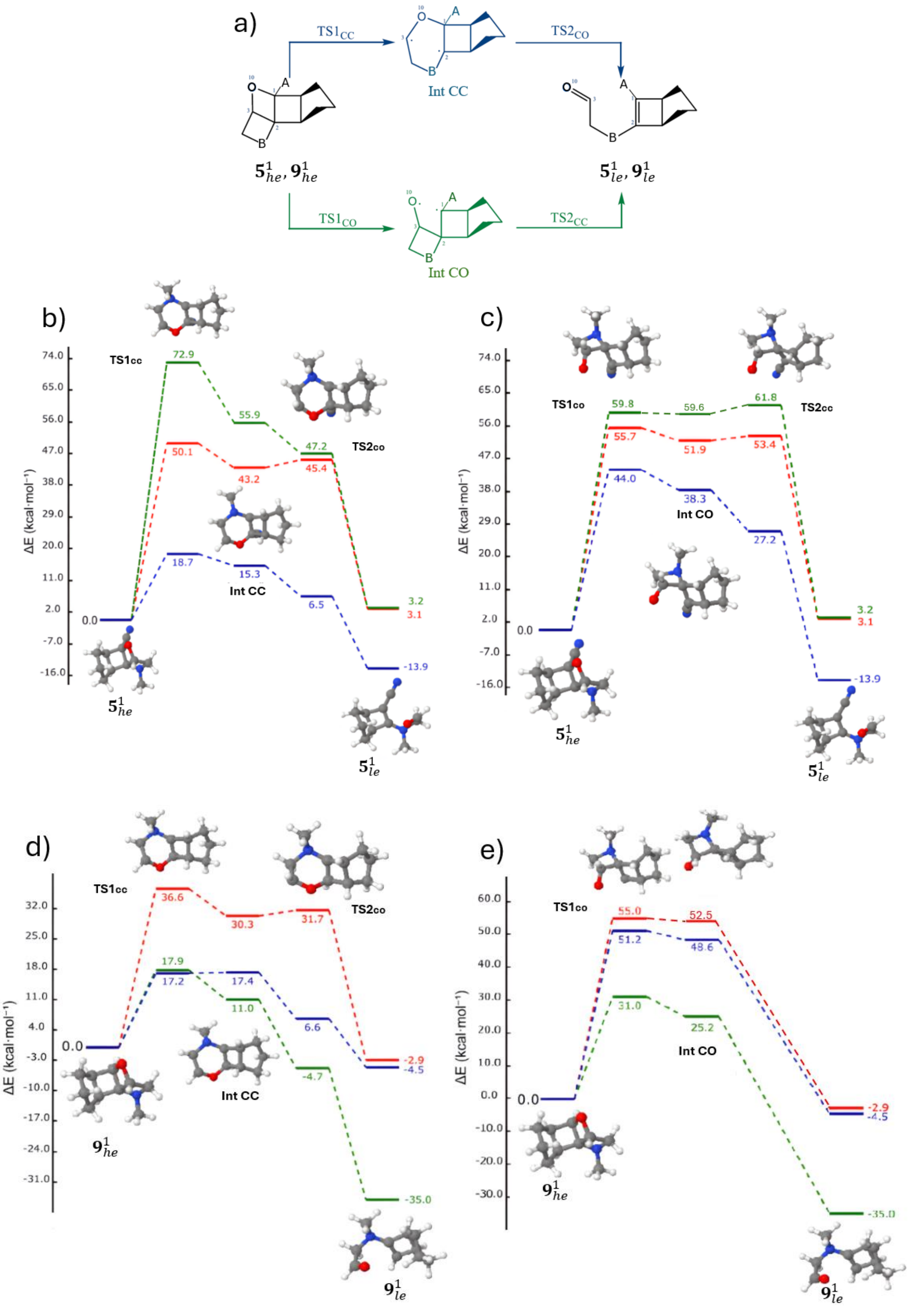

Figure S7. a) Scheme depicting the two possible thermal routes to release the stored chemical energy for $\mathbf{5}^1_{he}$ and $\mathbf{9}^1_{he}$: breaking first the C-C (blue pathway) or the C-O (green pathway) σ-bond, including the formation of the corresponding diradical intermediate. The energetics of the two routes are shown in b) and c) for $\mathbf{5}^1_{he}$, and in d) and e) for $\mathbf{9}^1_{he}$, including the structures of each saddle point. The energy profiles are given at CASSCF/6-31G(d) (red), CASSCF/ANO-S//CASSCF/6-31G(d) (green), and CASPT2/ANO-S//CASSCF/6-31G(d) (blue) level of theory.

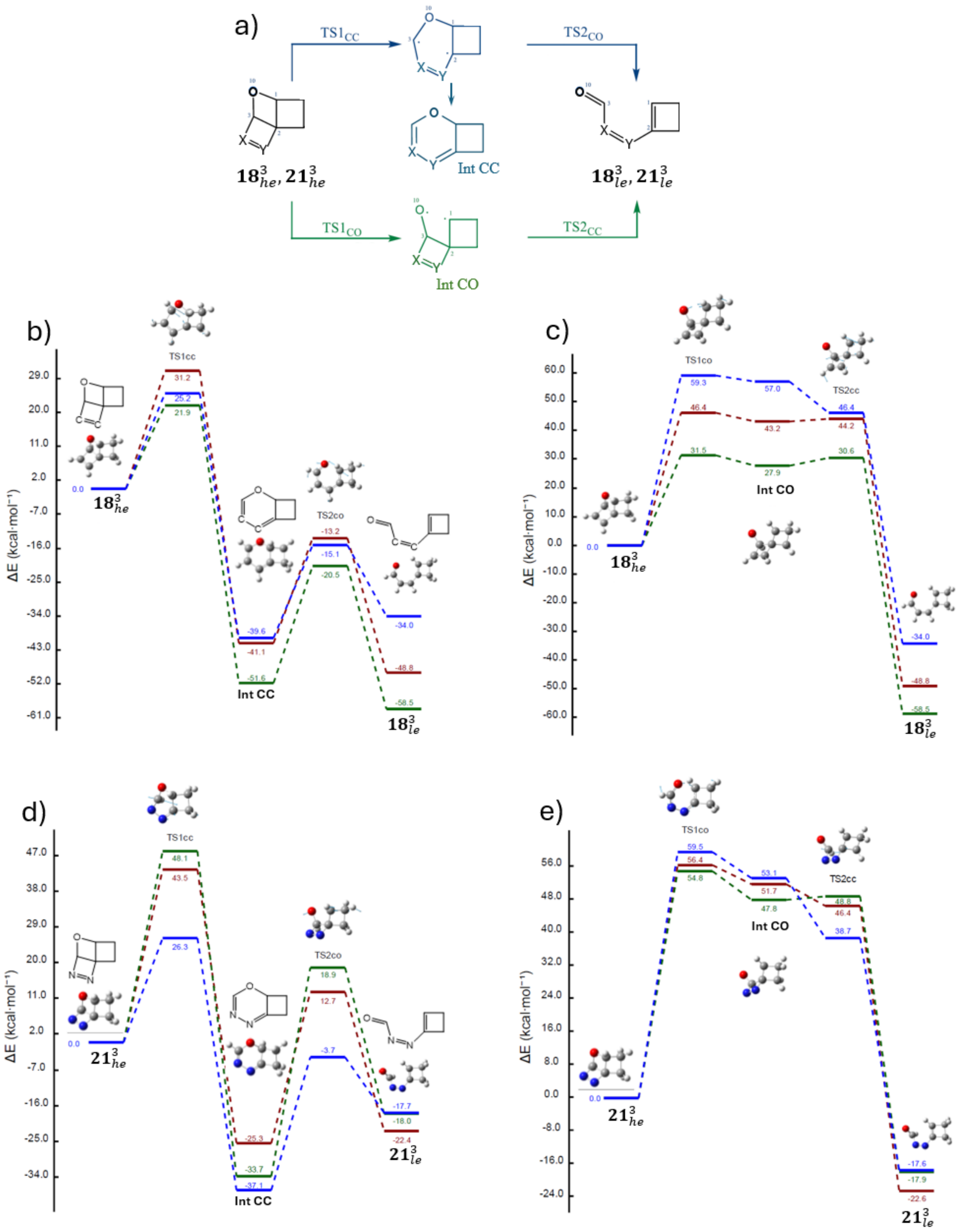

Figure S8. Scheme depicting the two possible thermal routes to release the stored chemical energy for $\mathbf{18}^3_{he}$ and $\mathbf{21}^3_{he}$: breaking first the C-C (blue pathway) or the C-O (green pathway) σ-bond, including the formation of the corresponding diradical intermediate. The energetics of the two routes are shown in b) and c) for $\mathbf{18}^3_{he}$, and in d) and e) for $\mathbf{21}^3_{he}$, including the structures of each saddle point (the transition state arrows indicate the direction dictated by the imaginary frequency). The energy profiles are given at CASSCF/6-31G(d) (red), CASSCF/ANO-S//CASSCF/6-31G(d) (green), and CASPT2/ANO-S//CASSCF/6-31G(d) (blue) level of theory.

Table S2. Electronic ground state energies of 3rd generation compounds with X=Y=CH (**18**^{3}) and X=Y=N (**21**^{3}). Both low-energy (*le*) isomers and intermediate along the C-C bond breaking pathway (Int CC) are considered. The "CASPT2//CASSCF energy" refers to the CASPT2 energy correction of a CASSCF fully optimized structure, *i.e.*, MS(5)-CASPT2/ANO-S//CASSCF-6-31G(d) level. The "CASPT2 energy" refers to a CASPT2 fully optimized structure, *i.e.*, MS(5)-CASPT2/ANO-S level. Active spaces: (10,8) for **18**^{3} and (14,10) for **21**^{3}.

Compound	CASPT2//CASSCF energy		CASPT2 energy		CASPT2//CASSCF – CASPT2
	**E** (Hartrees)	**ΔE** (Kcal·mol^{-1})	**E** (Hartrees)	**ΔE** (Kcal·mol^{-1})	**ΔE** (Kcal·mol^{-1})
$\mathbf{18}^3_{le}$	-345,890408	0,000000	-345,890388	0,000000	-0,012318
$\mathbf{18}^3_{\text{Int CC}}$	-345,899384	-5,632377	-345,899383	-5,644400	-0,000295
$\mathbf{21}^3_{le}$	-377,909359	0,000000	-377,909260	0,000000	-0,061802
$\mathbf{21}^3_{\text{Int CC}}$	-377,940420	-19,490702	-377,940419	-19,552247	-0,000257

Table S3. CASPT2 $S_0 \rightarrow S_1$ maximum absorption wavelength (λ_{abs}), oscillator strength (*f*), and energy-storage density (ρ_s) calculated for **18**^{3} substituted compounds (see Figure 6). Since substitution in *para* position to the oxygen results in consistent absorption red-shifts compared to *meta* substitutions, the attention was focused on **18**^{3}-c and **18**^{3}-d compounds.

Compound	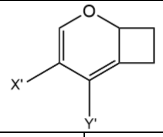		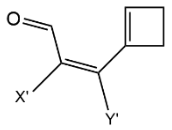		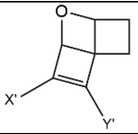		ρ_s (kJ g^{-1})
	λ_{abs} (nm, eV)	*f*	λ_{abs} (nm, eV)	*f*	λ_{abs} (nm, eV)	*f*	
**18**^{3}-a	351, 3.53	1.06·10^{-1}					
**18**^{3}-b	377, 3.29	8.91·10^{-2}					
**18**^{3}-c	361, 3.44	2.82·10^{-2}	360, 3.44	1.25·10^{-1}	238, 5.21	3.18·10^{-2}	0.83
**18**^{3}-d	402, 3.08	5.81·10^{-2}	412, 3.01	1.45·10^{-3}	271, 4.58	3.74·10^{-1}	0.90

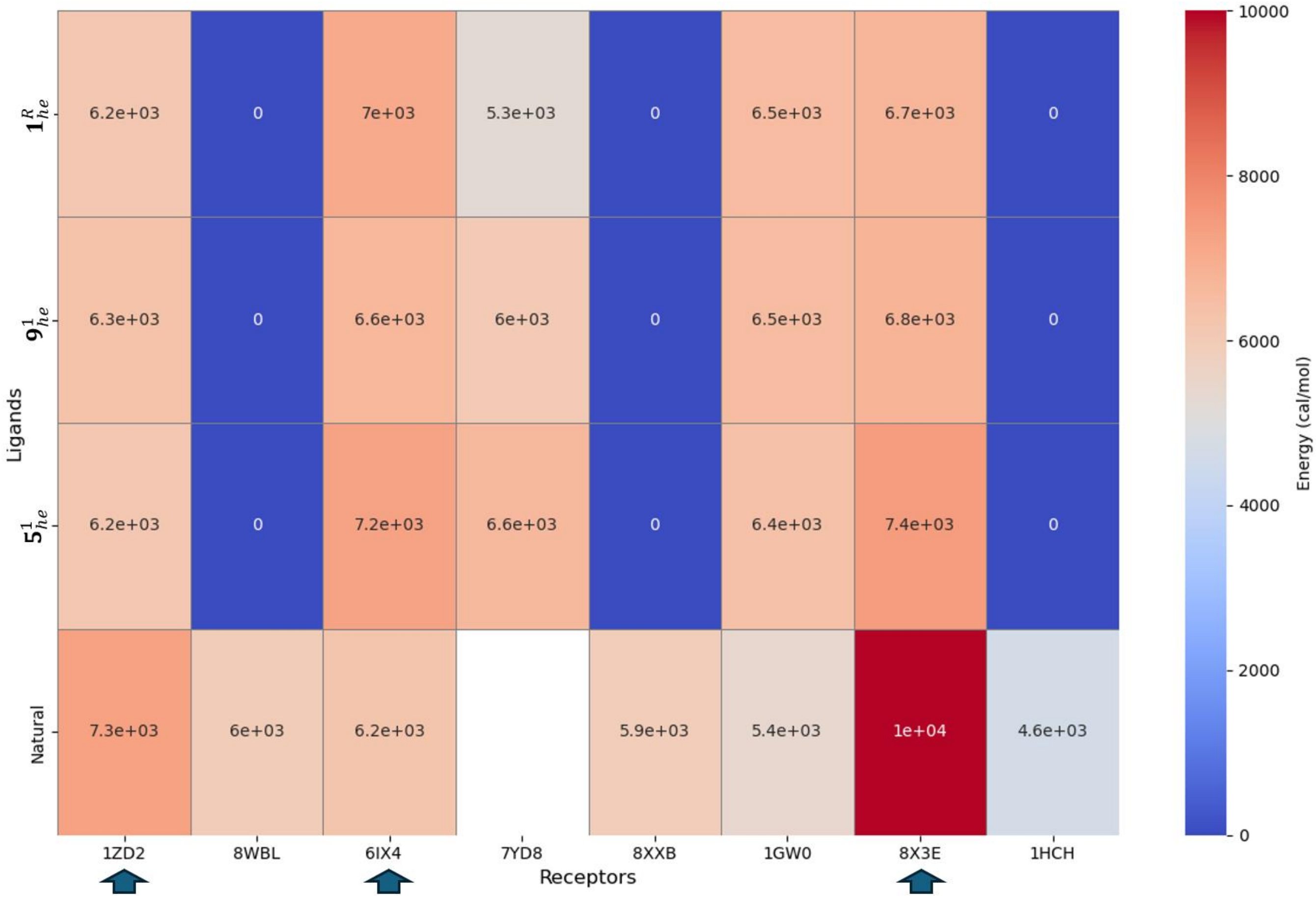

Figure S9. Heatmap depicting the highest interaction energy (cal/mol) calculated for all the receptor (PDB code)-ligand combinations considered in this work, within the active site. The three selected receptors, showing as a whole the highest interaction energies for all ligands, are indicated by an arrow. The interaction between the receptor 7YD8 and its natural ligand was not calculated due to the complexity of the natural ligand (DNA strand containing a thymine dimer, see Table 4) and the absence of its crystal structure.

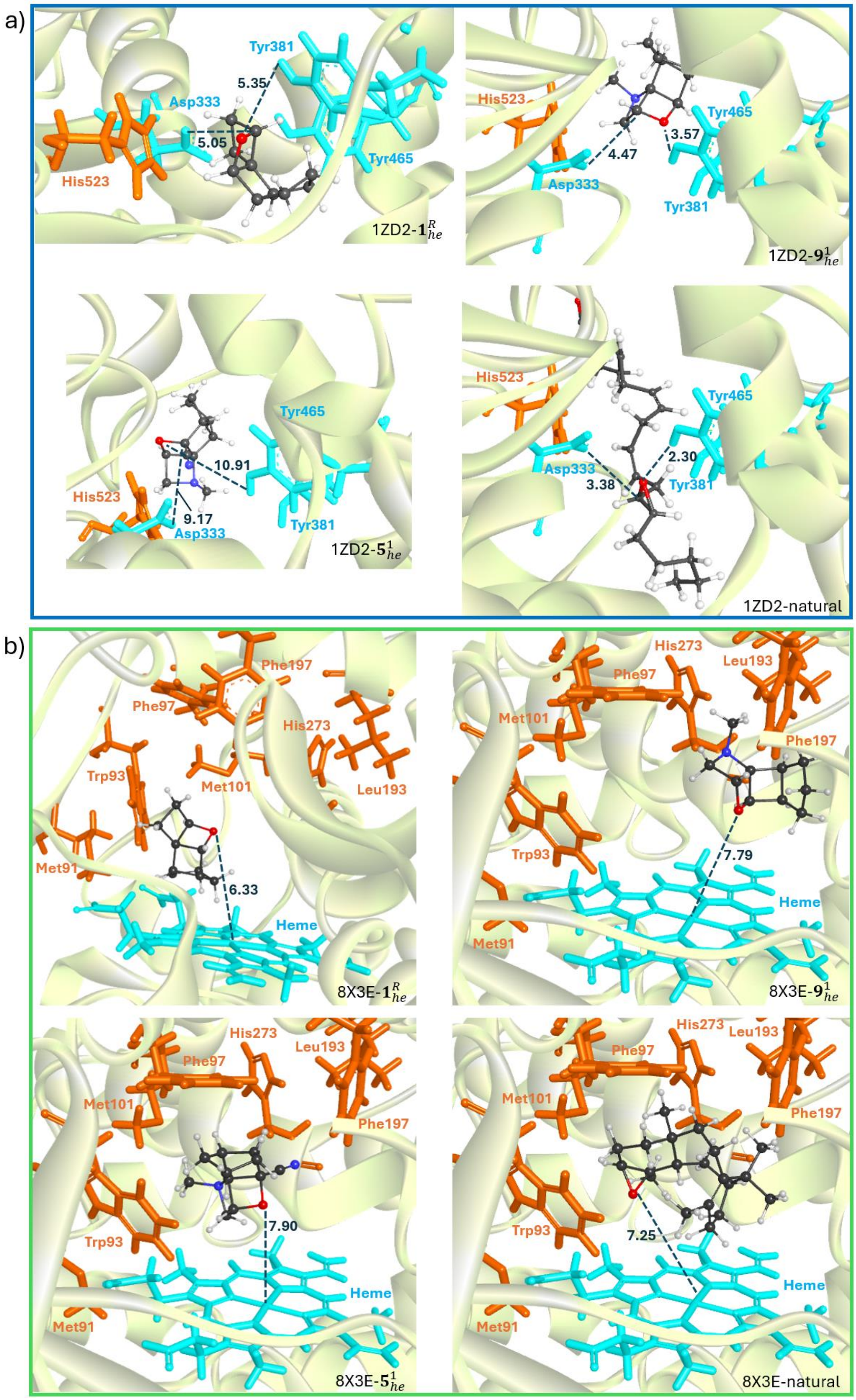

Figure S10. The structure and orientation of representative poses, corresponding to the highest interaction energies for a) human epoxide hydrolase and b) *Taxus cuspidata* taxadiene-5α-hydroxylase are shown (within blue and green frames, respectively, following the color code of Figure 7a). The most relevant distances between the ligand and the surrounding active site are indicated in Ångström (color code: cyano when directly involved in the catalytic reaction; orange when found as relevant but not directly involved).

Table S4. Species (biological origin), enzymatic type, Protein Data Bank (PDB) code, residues composing the enzymatic active site, and natural ligand involved in the catalytic activity for all receptors considered in this study.

Species	***Type***	***PDB code***	***Active site***	***Natural ligand***
Human	Soluble epoxide hydrolase	1ZD2	Asp333, Tyr381, Tyr465, His523	14,15-epoxyeicosa-5,8,11-trienoic acid
Rhodococcus opacus	*cis*-epoxysuccinate hydrolases	8WBL	Asp18, His190, Glu212	*cis*-epoxysuccinic acid
Aspergillus usamii	Epoxide hydrolase	6IX4	Asp191, Tyr249, Tyr312	*o*-nitrostyrene oxide
Methanosarcina mazei	class II CPD photolyase	7YD8	FAD, Arg256, Trp421, Trp305	DNA strand containing a thymine dimer
Thauera aminoaromatica S2	halohydrin dehalogenase	8XXB	Ser117, Tyr130, Arg 134	RAC1B
Melanocarpus albomyces	Laccase	1GW0	Ile 64, Met 65, Tyr 145, Gly 148, Val 150, Thr 173, Asp 174, Tyr 175, Tyr 176, Thr 232, Ser 233, Thr 234, Leu 299, Gly 346, Cu	2-metoxiphenol
Taxus cuspidata	Taxadiene-5alpha-hydroxylase	8X3E	Hemo, Met 91, Trp93, Phe 97, Met 101, Leu 193, Phe 197, His273	Taxa-4,11-diene oxide
Horseradish	Peroxidase	1HCH	Arg 38, His 42, His 170, heme	2-metoxiphenol

Table S5. Position and size of the box selected for performing molecular docking, considering each enzyme as a potential receptor. All values are given in Ångström. X, Y, and Z refer to Cartesian coordinates.

Receptor (PDB code)	Center X	Center Y	Center Z	Size X	Size Y	Size Z
1ZD2	-19.304	-3.258	62.354	40	36	52
8WBL	-29.000	-11.972	-21.778	34	36	22
6IX4	-4.769	1.831	59.482	30	28	24
7YD8	-14.844	15.778	0.062	20	20	20
8XXB	41.940	-46.552	7.761	20	20	20
1GW0	-0.337	-4.779	0.000	52	38	40
8X3E	74.521	65.676	34.786	14	14	14
1HCH	0.000	3.098	9.312	38	36	36

Table S6. Cartesian coordinates (in Ångström) of the most relevant structures.

$\mathbf{1}_{le}^{R}$

0 1			
C	2.63536900	0.87561200	-0.42466000
C	2.64552000	-0.59936800	-0.10867000
C	1.49603000	-1.01639900	0.79896000
H	2.62874100	-1.15778800	-1.05421000
H	3.62103000	-0.82337700	0.34811000
C	0.17798000	-0.95649000	0.10226000
H	1.66905100	-2.03274900	1.17840000
H	1.46937000	-0.35808900	1.67589000
C	-0.22002000	-0.81289000	-1.18170000
C	-1.69540000	-0.79843100	-0.84717000
C	-1.23668000	-0.98001000	0.65073000
C	-2.36814100	0.57745900	-0.90876000
H	-2.29780900	-1.60767100	-1.27706000
C	-1.66916000	0.29546900	1.38053000
H	-1.54394900	-1.90755100	1.15009000
C	-1.76419100	1.35211900	0.27116000
H	-3.44884100	0.45968800	-0.75603000
H	-2.22366100	1.07726900	-1.87349000
H	-2.36946200	2.21666900	0.56236000
H	-0.76344100	1.71651000	0.02111000
H	-2.65811000	0.13815900	1.83038000
H	-0.98176100	0.58119000	2.18513000
H	0.30856000	-0.67527900	-2.12497000
O	1.90083900	1.69305200	0.11514000
H	3.37571900	1.20617300	-1.18295000

$\mathbf{1}_{he}^{R}$

0 1			
C	-0.25461100	-0.93209900	-0.53056700
C	-0.61356500	0.39294800	0.18754300
C	-1.88077500	-0.39514400	0.57052800
C	-2.68505700	0.55315500	-0.35517200
C	-1.45150500	1.50081300	-0.44569000
H	-3.59371500	0.98799200	0.07099900
H	-2.93477500	0.05177600	-1.29387300
H	-1.55363300	2.36266200	0.22360600
H	-1.15411600	1.86377000	-1.43538900
O	-1.44054000	-1.62874200	-0.05355400
H	-2.24318400	-0.54164300	1.59485200
C	0.98069800	-1.06481500	0.38508900
H	-0.10300500	-1.05561000	-1.61180100
C	0.66576100	0.34539600	1.01850900
C	2.30429500	-0.89627800	-0.36079000
H	0.93507700	-1.94000200	1.04058300
C	2.33864800	0.59259800	-0.72860200
H	3.14058400	-1.12915700	0.31022800
H	2.38128400	-1.56154500	-1.22878400
C	1.78189500	1.27469700	0.52681000
H	0.51563800	0.40218600	2.10327500
H	1.42473600	2.29489800	0.34390700
H	2.56849100	1.33493000	1.28943100
H	3.34028200	0.94477900	-0.99578900
H	1.68595200	0.78727500	-1.58877500

$\mathbf{2}_{le}^{1}$ (A: $-NH_2$; B: -H)

0 1			
C	-2.52971400	-0.62141000	-0.82371000
C	-2.43202400	0.71211800	-0.12856600
C	-1.56871000	0.67241500	1.13393900
H	-2.04882900	1.44056900	-0.85500700
H	-3.46388800	1.02551800	0.09545200
C	-0.10667900	0.61014700	0.81752100
H	-1.79188900	1.55712100	1.74729700
H	-1.85102100	-0.20198600	1.72959200
C	0.71661700	1.34916100	0.02936000
C	1.94972600	0.53035600	0.30533300
C	1.01479400	-0.31932700	1.24992700
C	2.39251500	-0.41793600	-0.81441700
H	2.77853100	1.06487600	0.78926700
C	0.99322400	-1.72998700	0.65395000
H	1.27254800	-0.29467900	2.31713200
C	1.32129000	-1.51710500	-0.82961400
H	3.36461800	-0.85474500	-0.55211400
H	2.50626200	0.08803400	-1.78016300
H	1.66265900	-2.43199200	-1.32494600
H	0.43020800	-1.17119200	-1.36124600
H	1.78138900	-2.33558100	1.12139500
H	0.03655800	-2.23822700	0.80618500
O	-2.15775400	-1.68429500	-0.34226800
H	-3.00402600	-0.60198000	-1.82728000
N	0.55400300	2.40614700	-0.87215500
H	-0.20449500	3.03532400	-0.62244100
H	1.40915500	2.93967400	-1.00223300

$\mathbf{3}_{le}^{1}$ (A: $-NH_2$; B: $-NH_2^+$)

1 1			
C	-2.62351300	-0.45922600	-0.83081100
C	-2.19728900	0.91936700	-0.34529200
H	-1.52695500	1.41106600	-1.05476900
H	-3.08212500	1.54692400	-0.18762000
C	-0.01725600	0.57029200	0.79809300
H	-1.71999700	1.44407900	1.63816200
H	-1.83473100	-0.19237700	1.32276700
C	0.88586700	1.31569000	0.11679800
C	1.98967200	0.32359900	0.41029700
C	0.92378600	-0.52507300	1.22086400
C	2.40055300	-0.55929200	-0.77438000
H	2.82189500	0.72828100	0.99647100
C	0.80338800	-1.85956100	0.48041300
H	1.11533900	-0.61141600	2.29596600
C	1.23222900	-1.53714100	-0.95625300
H	3.30462200	-1.11307000	-0.49844700
H	2.63101100	0.01731800	-1.67640000
H	1.51694000	-2.42895700	-1.52072000
H	0.41403600	-1.05772300	-1.50680600
H	1.50844700	-2.57101900	0.92557800
H	-0.19394100	-2.30695600	0.54748300
O	-2.53879400	-1.42626100	-0.08646100
H	-3.02537500	-0.53112100	-1.85192400
N	-1.47630500	0.71311700	0.95543400
N	0.89769000	2.42792000	-0.67704600
H	0.23062400	3.16845300	-0.49022200
H	1.81624600	2.78740900	-0.91492800

$\mathbf{4}_{le}^{1}$ (A: -CN; B: -NH)

0 1			
C	2.43478200	-0.80428400	0.86637400
C	2.43079200	-0.53841600	-0.62933100
H	2.61423800	0.52787100	-0.80382300
H	3.27200500	-1.07664200	-1.08578600
C	0.07756700	-0.18680500	-0.97619400
H	1.01912600	-1.92909000	-1.26806400
C	-0.15751300	1.06382900	-0.48198400
C	-1.66806100	0.91598300	-0.57830500
C	-1.37962200	-0.53122000	-1.13997300
C	-2.39267900	0.71277800	0.75313900
H	-2.16601900	1.60856600	-1.26629500
C	-1.93167700	-1.50249600	-0.08836900
H	-1.70666700	-0.73676300	-2.16593200
C	-1.95976900	-0.68631200	1.21169300
H	-3.47668000	0.72913000	0.58114900
H	-2.16010500	1.49879900	1.48012500
H	-2.63603300	-1.11354900	1.95871800
H	-0.95882800	-0.65584000	1.65143400
H	-2.95090500	-1.79230700	-0.37268300
H	-1.33903200	-2.42060600	-0.00087700
O	1.54155200	-1.41331900	1.43761000
H	3.31742400	-0.42480900	1.41814800
C	0.67718900	2.07866400	0.04251400
N	1.38661700	2.92183200	0.48513100
N	1.19238400	-0.92733600	-1.27147000

$\mathbf{5}_{le}^{1}$ (A: -CN; B: -N(Me))

0 1			
C	2.37303300	0.22928300	1.09873800
C	2.39418500	-0.03093000	-0.39922100
H	2.37968100	0.93409500	-0.92110600
H	3.34454100	-0.51532900	-0.66237400
C	0.05841200	-0.27204000	-0.80227800
C	-0.39494000	1.01463800	-0.68023500
C	-1.85351300	0.61245900	-0.80408400
C	-1.32318500	-0.87086700	-0.92473900
C	-2.65838500	0.67121800	0.49507900
H	-2.37874500	1.00680700	-1.68180700
C	-1.84622100	-1.58998100	0.32727500
H	-1.53625700	-1.39103700	-1.86651000
C	-2.08102600	-0.46695800	1.34594700
H	-3.71634300	0.47383000	0.27825900
H	-2.59537500	1.65174800	0.97992800
H	-2.75011000	-0.77082900	2.15715900
H	-1.12907300	-0.16731900	1.79462600
H	-2.80072000	-2.07181200	0.07924800
H	-1.17002000	-2.36771200	0.69673300
O	1.56750600	-0.28816000	1.85881300
H	3.15041800	0.92649400	1.47080400
C	0.23382200	2.25103500	-0.41042100
N	0.77309400	3.28404400	-0.17874000
N	1.29138000	-0.85289600	-0.84392100
C	1.41099300	-2.28895100	-0.62551000
H	0.57493000	-2.79257000	-1.11271400
H	2.33895400	-2.63788700	-1.08760400
H	1.41770300	-2.54467400	0.44018100

$\mathbf{6}_{le}^{1}$ (A: -CN; B: -N(Et))

0 1			
C	1.87458600	1.42384900	0.93736000
C	2.01273100	1.02168300	-0.52459800
H	1.66882300	1.86353200	-1.14171300
H	3.07802100	0.88080400	-0.75281800
C	-0.07082900	-0.10426500	-0.82157900
C	-0.96721600	0.91985200	-0.65486400
C	-2.17394100	0.00649800	-0.76243000
C	-1.13559800	-1.16891800	-0.95664500
C	-2.89047700	-0.27984200	0.55938300
H	-2.84442200	0.20008600	-1.60797600
C	-1.32256200	-2.08008200	0.26328800
H	-1.16761400	-1.69155300	-1.92033900
C	-1.90245200	-1.15530200	1.34132100
H	-3.81111000	-0.84469300	0.36239100
H	-3.16963800	0.63841200	1.08776400
H	-2.37965900	-1.70811600	2.15668400
H	-1.10644000	-0.54134500	1.77470700
H	-2.05434900	-2.85790900	0.00944000
H	-0.40208200	-2.58397900	0.57566700
O	1.34140600	0.72588400	1.78598200
H	2.29933700	2.41595200	1.18971400
C	-0.82787200	2.29271000	-0.35489300
N	-0.68982100	3.44552100	-0.10165300
N	1.28349300	-0.17713400	-0.86293200
C	1.98249100	-1.45666600	-0.90576300
H	1.26891100	-2.19623700	-1.27814500
H	2.78330900	-1.38029300	-1.65296200
C	2.55850400	-1.89598400	0.43661100
H	3.03475600	-2.87637100	0.33293500
H	3.31544900	-1.19168700	0.79166000
H	1.77556000	-1.95359100	1.19407500

$\mathbf{7}_{le}^{1}$ (A: -CN; B: $-NH_2^+$)

1 1			
C	2.57317200	-0.40085200	0.81330500
C	2.42276100	-0.70130000	-0.67476000
H	2.47201700	0.19191900	-1.29972900
H	3.19694800	-1.40604000	-0.99809800
C	-0.05841900	-0.42075100	-0.87087800
C	-0.14326800	0.89917100	-0.60101500
C	-1.66169000	0.87002000	-0.73762700
C	-1.52698600	-0.68540300	-1.01948000
C	-2.40343400	0.98670400	0.59577400
H	-2.05324500	1.47855700	-1.55762000
C	-2.18778200	-1.37806100	0.18148900
H	-1.85803400	-1.03771000	-2.00213400
C	-2.12235800	-0.34138300	1.31129100
H	-3.47520600	1.07937600	0.38786000
H	-2.10117900	1.86875400	1.16932300
H	-2.84379000	-0.54872800	2.10543600
H	-1.12947100	-0.33186800	1.77553100
H	-3.23166300	-1.58401300	-0.07911300
H	-1.72785400	-2.33966100	0.43880200
O	1.79335500	-0.90921000	1.60716300
H	3.40579900	0.24800200	1.11850400
C	0.79342300	1.89050000	-0.21378800
N	1.61612800	2.67736000	0.12214900
H	1.05621100	-1.96796000	-1.66093800
H	0.95670200	-1.94568500	0.01532000
N	1.08007200	-1.35495000	-0.83305300

$\mathbf{8}_{le}^{1}$ (A: -NH$_2$; B: -NH)

0 1

C	-2.53765300	-0.21302700	-0.88548000
C	-2.54312500	-0.22076100	0.63012800
H	-2.66485600	0.81162300	0.97096500
H	-3.42142400	-0.78540200	0.97301900
C	-0.17863500	-0.07877000	0.93350800
H	-1.24235000	-1.77294700	1.04623900
C	0.12571200	1.13098800	0.40188100
C	1.62497100	0.93096200	0.53955800
C	1.25953100	-0.47186700	1.16193100
C	2.36764200	0.62976300	-0.76654600
H	2.15682700	1.62463100	1.20486400
C	1.81178800	-1.51484600	0.18315300
H	1.55089200	-0.63423900	2.20665600
C	1.88875200	-0.77520200	-1.15960000
H	3.45022700	0.61608300	-0.58473300
H	2.17830200	1.38140900	-1.54324200
H	2.55855400	-1.26602800	-1.87325600
H	0.89405300	-0.73007900	-1.61286200
H	2.81840700	-1.81336200	0.50294700
H	1.20109000	-2.42463500	0.13411900
O	-1.73420400	-0.83701200	-1.56529700
H	-3.34295700	0.38509200	-1.35986300
N	-1.34386900	-0.77608900	1.22776700
N	-0.68874800	2.13452700	-0.20421900
H	-0.53288800	3.03890900	0.24335900
H	-0.41582600	2.25253500	-1.18185900

$\mathbf{9}_{le}^{1}$ (A: -H; B: -N(Me))

0 1

C	-2.42174300	-1.29463700	0.27651000
C	-2.39568900	-0.18157400	-0.75707600
H	-2.23458800	-0.63191300	-1.74707900
H	-3.39482900	0.27792700	-0.78860500
C	-0.09682900	0.41345900	-0.74560600
C	0.47616700	-0.62497400	-1.40763900
C	1.88170200	-0.13190100	-1.14550800
C	1.21213200	1.06854900	-0.36643800
C	2.68006200	-0.91490100	-0.09824500
H	2.46840700	0.14829400	-2.02967300
C	1.65255900	0.90492000	1.09415700
H	1.38310600	2.07148300	-0.77729700
C	1.98358400	-0.58701800	1.22859500
H	3.71450400	-0.54701500	-0.07127900
H	2.71575600	-1.98892200	-0.31388000
H	2.60752400	-0.80296300	2.10205600
H	1.05836600	-1.16255100	1.32788900
H	2.56100600	1.49987200	1.25657800
H	0.90383800	1.24289500	1.81806200
O	-1.70915700	-1.32860100	1.26934300
H	-3.16338600	-2.09960800	0.08040700
N	-1.39758400	0.82924900	-0.52287600
C	-1.65387100	1.74114400	0.58409800
H	-0.90457200	2.53451200	0.56635000
H	-2.63718900	2.19916000	0.43779300
H	-1.63059200	1.24592800	1.56223800
H	0.10054900	-1.52144300	-1.89526700

$\mathbf{18}_{le}^{3}$ (X: -CH; Y: -CH)

0 1

C	-2.43901200	-0.27979800	-0.00008600
C	0.69858900	0.37986600	0.00025500
C	0.96208300	-0.95870000	-0.00018700
C	2.45880400	-0.77343000	-0.00032000
C	2.17282000	0.76003900	0.00032200
H	2.52284200	1.29371900	-0.89012300
O	-1.87822600	-1.37704400	0.00035700
H	-3.54653100	-0.23261800	-0.00038900
H	0.32915100	-1.83675900	-0.00021900
H	2.98243300	-1.14459000	0.88803300
H	2.52282000	1.29282400	0.89131500
C	-0.43782000	1.27448000	-0.00001700
H	-0.15924700	2.33017900	-0.00009800
H	2.98200600	-1.14370700	-0.88930400
C	-1.77555600	1.02653000	-0.00022300
H	-2.44711400	1.88337700	-0.00053200

$\mathbf{18}_{he}^{3}$ (X: -CH; Y: -CH)

0 1

C	0.65959700	-0.60030600	-0.73574600
C	-0.04177800	0.61903800	-0.05762100
C	-0.77199300	-0.51622100	0.71941400
O	-0.14256600	-1.58940900	-0.06616000
H	-0.68586700	-0.66867500	1.79970100
C	1.94805100	-0.17257900	0.01688800
C	1.29382700	1.19174400	0.39182600
H	0.72607000	-0.76659400	-1.81594700
H	1.63523800	1.99759400	-0.26688100
H	1.37374800	1.53494000	1.42853300
H	2.13764300	-0.82810500	0.87050700
H	2.85249400	-0.11407100	-0.59501300
C	-1.40102700	1.09606400	-0.44953100
H	-1.74173200	1.90629400	-1.09081400
C	-2.03710000	0.04661400	0.13291800
H	-3.05452000	-0.33223700	0.09030200

$\mathbf{19}^{3}_{le}$ (X: -CH; Y: -N)				$\mathbf{19}^{3}_{he}$ (X: -CH; Y: -N)			
0 1				0 1			
C	-2.29061800	0.27039200	-0.26471500	C	-0.62168800	0.62825600	-0.71488400
C	0.62817100	-0.40347700	0.09643800	C	0.04455100	-0.59524900	-0.03935800
C	0.93556600	0.88992800	0.39898900	C	0.80074900	0.50269200	0.74406000
C	2.37433300	0.73707100	-0.03796200	O	0.20862100	1.59607700	-0.03331400
C	2.02727400	-0.74843500	-0.37287100	H	0.78599700	0.66258000	1.82561100
H	2.54772600	-1.49027500	0.24068500	C	-1.91674500	0.20716700	0.03316700
O	-1.79751800	1.38285900	-0.09124000	C	-1.29254700	-1.18978400	0.34618200
H	-3.27401500	0.15922800	-0.76529000	H	-0.67767100	0.79380300	-1.79445200
H	0.38744500	1.69899100	0.86566700	H	-1.61168000	-1.94446000	-0.37982300
H	2.67699600	1.35451700	-0.89051500	H	-1.40416100	-1.59697000	1.35540600
H	2.11876500	-1.02753100	-1.42749600	H	-2.07784300	0.82953300	0.91664600
H	3.12015100	0.84834400	0.75685500	H	-2.82750200	0.19914200	-0.57079900
C	-1.66727300	-1.01715300	0.14420100	C	1.96207200	-0.17074600	0.06845800
H	-2.36791200	-1.84592700	0.28240900	H	3.02034000	0.06707400	-0.04201400
N	-0.41053200	-1.30431200	0.26901800	N	1.32360000	-1.15333300	-0.52425900

$\mathbf{20}^{3}_{le}$ (X: -N; Y: -CH)				$\mathbf{20}^{3}_{he}$ (X: -N; Y: -CH)			
0 1				0 1			
C	0.89294000	-0.44666900	-0.36683500	C	0.62876200	-0.61107500	-0.71870300
C	0.23896600	0.73636000	-0.21878600	C	-0.02612400	0.63711400	-0.04135300
C	-2.08046500	-0.95917100	0.20735300	C	-0.81019500	-0.47222600	0.69999300
O	-1.71821600	-1.85001600	0.96103100	O	-0.19736300	-1.56501500	-0.01907600
H	-2.54825200	-1.16240300	-0.77439000	H	-0.87138400	-0.61598800	1.78203000
C	2.11361900	0.19038700	-0.98461900	C	1.93938600	-0.20987100	0.00570800
C	1.37873200	1.55735300	-0.79602200	C	1.33331300	1.18077200	0.36471300
H	0.65382500	-1.47090100	-0.09734500	H	0.66413300	-0.78836500	-1.79780700
H	1.15687700	2.09845000	-1.72158700	H	1.68159400	1.96086400	-0.31997400
H	1.86704800	2.24058200	-0.09347400	H	1.44418200	1.54279800	1.39139800
H	2.31472100	-0.08416100	-2.02565800	H	2.11897900	-0.85532900	0.86886100
H	3.04038400	0.08556600	-0.41044700	H	2.83543100	-0.19166000	-0.62017800
C	-1.05215200	1.14778900	0.32211900	C	-1.39856200	1.04469000	-0.42058700
H	-1.16137600	2.20616600	0.57579600	H	-1.81856100	1.84624200	-1.03088600
N	-2.08611600	0.40442800	0.52295500	N	-2.06784900	0.02930600	0.07721900

$\mathbf{21}^{3}_{le}$ (X: -N; Y: -N)				$\mathbf{21}^{3}_{he}$ (X: -N; Y: -N)			
0 1				0 1			
C	-1.84966400	0.21474100	-0.47193400	C	-0.58302200	0.63671600	-0.70023800
C	0.67241500	-0.41513300	0.10665400	C	0.02719800	-0.61648500	-0.00904000
C	0.79021700	0.92171600	0.33879300	C	0.84713200	0.45360100	0.71754900
C	2.25513900	0.92769200	-0.03698200	O	0.27336500	1.56647300	0.00680600
C	2.12239800	-0.61468000	-0.26536900	H	0.99513400	0.60429800	1.78995900
H	2.69348000	-1.22754500	0.43763900	C	-1.90574000	0.26288300	0.01803400
O	-2.48999000	1.06827200	0.11129700	C	-1.34253200	-1.15787600	0.33754000
H	-1.56671800	0.27424300	-1.54139400	H	-0.59824300	0.79912600	-1.78099100
H	0.12501900	1.67997600	0.74057000	H	-1.67249600	-1.89458500	-0.40137800
H	2.50916200	1.51272600	-0.92660700	H	-1.49270000	-1.56603500	1.34059800
H	2.31139500	-0.96269100	-1.28511100	H	-2.05843700	0.88810900	0.90066500
H	2.93878300	1.20102800	0.77336200	H	-2.80322800	0.29303100	-0.60429000
N	-0.32156700	-1.39157000	0.30495700	N	1.30440700	-1.14226200	-0.49748600
N	-1.54046800	-1.06984900	0.10707000	N	2.00771200	-0.16184700	0.00004500
